# Paradigm shift for *cry* gene expression in *Bacillus thuringiensis*

**DOI:** 10.1128/mbio.01592-26

**Published:** 2026-08-05

**Authors:** Emilie Verplaetse, Alicia Nevers, Leyla Slamti, Didier Lereclus

**Affiliations:** 1 Université Paris-Saclay, INRAE, AgroParisTech, Micalis Institute 301985, Jouy-en-Josas, France; The University of Texas Health Science Center at Houston, Houston, Texas, USA

**Keywords:** biopesticide, plasmid, gene regulation, transcription, insecticidal, noctuidae

## Abstract

In most *Bacillus thuringiensi*s strains, the *cry* genes are transcribed by RNA polymerases containing sporulation-sigma factors E or K, leading to the formation of an insecticidal crystal within the mother cell along spore development. The *kurstaki* HD1 strain, a parent of commercial strains, also releases the insecticidal proteins Cry1I and Vip3A in the extracellular medium. *vip3A* expression is activated by the transcriptional regulator VipR at the onset of the stationary phase. Here, we expanded the VipR regulon in strain HD1 by identifying the VipR-binding box upstream from the *cry2Aa*, *cry2Ab*, and *cry1Ia* genes, and conducting transcription assays. Unexpectedly, a VipR box was located in the promoter of a putative N-acetylmuramoyl-l-alanine amidase (*ami*) gene upstream from *cry1Ac* in strain *kurstaki* HD73, closely related to the HD1 but devoid of *vipR*. Introduction of *vipR* in this strain led to the expression of the *ami-cry1Ac* operon, resulting in an early and increased production of Cry1Ac. We demonstrated that Cry1Ac was also produced in a VipR-dependent manner in an HD73 ∆*spo0A* mutant. Similarly, an HD1 ∆*spo0A* strain produces all the insecticidal proteins encoded in its genome, including *cry2Ab*, previously considered unexpressed. A genomic analysis also revealed the presence of putative VipR-binding sequences in lepidopteran-active strains, upstream from *cry* genes such as *cry1E*, *cry1F*, *cry9D*, and *cry9E*. Overall, our results break the dogma on the regulation of *cry1A* and *cry2A* genes and provide evidence of sporulation-independent Cry toxin production in biopesticidal Bt strains.

**IMPORTANCE:**

*Bacillus thuringiensis* is a remarkably efficient entomopathogen due to its ability to produce various insecticidal proteins, such as Cry or Vip. This property has made it a highly effective biopesticide used worldwide. Our work modifies the paradigm of *cry1* and *cry2* genes being regulated solely by sporulation-specific sigma factors and thus exclusively expressed during this process. Indeed, we demonstrated that the VipR regulator controls the transcription of *vip3Aa*, *cry2Aa*, *cry2Ab*, *cry1Ia*, and the *ami-cry1A* operons encoded by a strain closely related to that of commercial biopesticides and specifically turns on their expression from the onset of the stationary phase, leading to the production of insecticidal crystals independently of sporulation. By providing new knowledge on the regulation of insecticidal protein genes, these findings bring new insight for the genetic improvement of Bt strains used as commercial biopesticides.

## INTRODUCTION

*Bacillus thuringiensis* (Bt) is a bacterium famous for its ability to produce a spore and massive amounts of insecticidal proteins that will form a parasporal crystal inclusion (1, 2). The pathogenic properties of the bacterium towards insects rely on the production of Cry and Cyt toxins that have the property to crystallize and on the production and release of Vip, Sip, and a few Cry toxins unable to form a crystal (3, 4). The majority of *cry*, as well as the *cyt* and *vip3* genes, are located on plasmids, and many Bt strains carry various combinations of insecticidal protein genes (1).

Since the first studies in the 1980s–1990s, a close association between *cry* gene expression and the sporulation process has been evidenced. Indeed, studies of *cry1A*, *cry4A*, *cry4B*, *cry11A*, *cry18*, and *cyt1A* gene expression have demonstrated that transcription of the insecticidal protein-coding genes was initiated from two overlapping promoters located upstream from the *cry* open reading frame (5–9), while *cry2* and *cry15* genes were transcribed from one promoter (10, 11). It was demonstrated that the *cry* and *cyt* promoters were transcribed by an RNA polymerase that contains the sporulation-specific sigma factor E (SigE) or K (SigK), active sequentially during sporulation, which allows a sustained production of toxins in the mother-cell compartment during this process (9, 12, 13). Thus, it is widely admitted that expression of the *cry* and *cyt* genes encoding insecticidal proteins active against lepidopteran and dipteran insects is exclusively sporulation-dependent (14). This is particularly true of commercially-used Bt strains such as *kurstaki* HD1 and *israelensis*.

Besides this established link between sporulation and *cry* gene expression, a few observations questioning this paradigm have been reported without any explanations. Notably, the transcription of *cry1A*, *cry1E*, *cry1I*, and *cry2A* started before the onset of sporulation, and a faint production of Cry1 and Cry2 proteins was detected in a time frame that precedes the activation of SigE and SigK, in Bt *kenyae* and *kurstaki* strains isolated in Mexico (15). While the *cry1Ac* gene of Bt *kurstaki* HD73 is a typical example of a SigE- and SigK-dependent crystal gene, it has been shown that a weak transcription of *cry1Ac* occurred in sporulation mutants of the HD73 strain (16). In a very different way from the genes encoding toxins active on Lepidoptera and Diptera (e.g*.*, *cry1*, *cry2*, and *cry4*), other modes of expression have been demonstrated for Bt strains active on other insect orders. The expression of the Cry3*-*coding gene is controlled by SigA and is independent of the sporulation sigma factors (17–19). Consequently, this coleopteran-specific pesticidal protein is produced from the end of the vegetative growth throughout the stationary phase (2). Finally, the atypical production of Cry proteins by the LM1212 and HD977 strains has been unraveled by identifying CpcR, a specific transcriptional regulator located on a large plasmid (20, 21). In the LM1212 strain, the regulator leads to the production of Cry proteins only in a subpopulation that will not form a spore in a division of labor strategy (22).

The regulation of the insecticidal proteins that do not crystallize and that are liberated in the growth medium, such as Vip3A (4) or Cry1I (23), has remained unknown until the identification of VipR, the transcriptional regulator required for the expression of *vip3A*, in the HD1 strain (24). Both the *vipR* and *vip3A* genes are located on the large conjugative plasmid pBMB299 of this strain. VipR belongs to the PCVR family, together with the *B. anthracis* AtxA and the *Streptococcus pyogenes* Mga (25). Proteins that belong to this family of virulence regulators share a similar structure rather than amino acid sequence homology. They contain a phosphoenolpyruvate phosphotransferase system (PTS)-regulatory domain(s), and their activity is therefore modulated by phosphorylation, most probably via the PTS as a response to the carbohydrate availability of their host environment. The putative DNA binding site of VipR, designated VipR box, is an imperfect palindromic sequence, present in the *vip3A* promoter and in the promoter of the *vipR* gene, which is autoregulated (24). The VipR box was also found in the promoter region of other transcription units on the pBMB299 plasmid. Among these are the *cry1I*, *cry2Ab*, and the three-gene operon harboring *cry2Aa*, suggesting that VipR may also be involved in expressing these *cry* genes. The VipR box was also identified in the promoter region of two N-acetylmuramoyl-l-alanine amidase-encoding genes or pseudogenes (24). Analysis of *vipR* and *vip3A* transcription in the wt and in a sporulation mutant showed that the expression of these genes is induced at the onset of the stationary phase (T0). Consequently, production of Vip3A was detected only after the T0.

The present study overturns the dogma that Cry1 and Cry2 insecticidal proteins are strictly produced during sporulation. We demonstrate an early production of Cry proteins independent of the sporulation genetic network, but VipR dependent, in Bt strains that produce a functional VipR regulator. In summary, our study shows that, upon entry into the stationary phase, VipR controls the expression of all the insecticidal protein genes in the *kurstaki* strain HD1. Genomic analysis suggests that this VipR-controlled Cry production mechanism is also present in other strains with anti-lepidopteran activity.

## RESULTS

### VipR controls the expression of all the insecticidal protein genes of the Bt *kurstaki* HD1 strain

**Fig 1:**
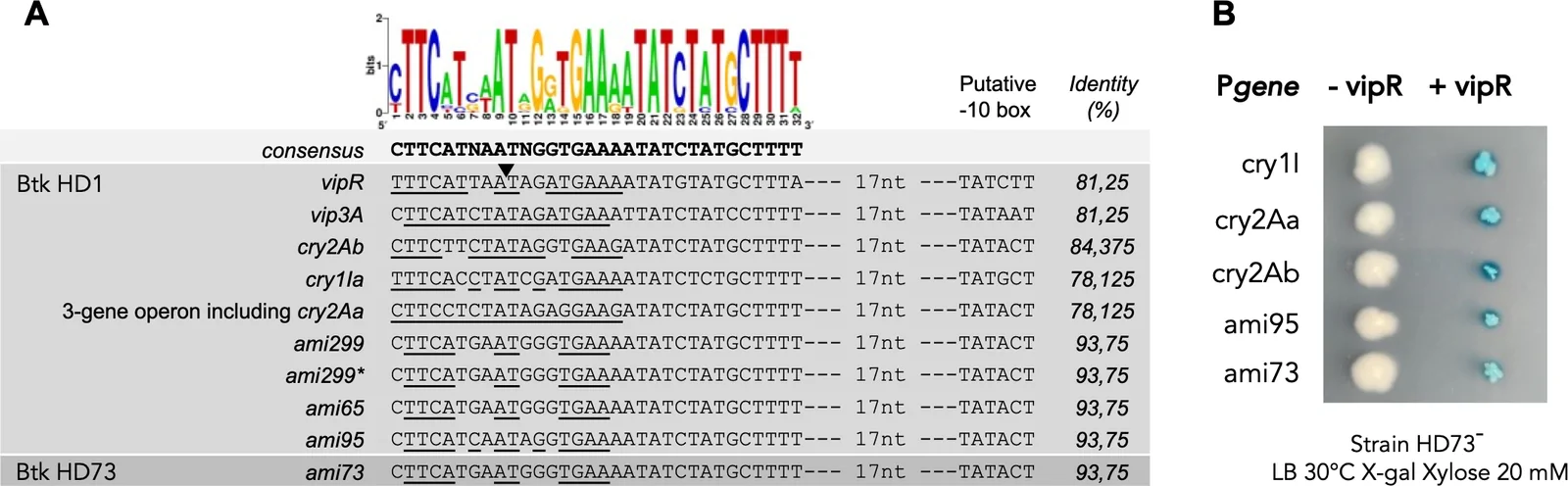
VipR controls the expression of the insecticidal protein genes containing a VipR box in their promoter. (**A**) Alignment of the VipR boxes present on the pBMB299, pBMB65, and pBMB95 of the HD1 strain and on the pHT73 of the HD73. The name of the gene that contains the conserved sequence in its promoter is indicated on the left. The distance between the putative −10 box and the last nucleotide of the box is indicated. A consensus sequence composed of base frequency with a threshold >50% in the 10 sequences used in the alignment is shown on top. Underlined nucleotides correspond to the palindrome (center is indicated with an arrowhead). Percentage of identity compared to the consensus sequence is indicated on the right. Figure adapted from reference 25. (**B**) The strains HD73^−^ harboring the plasmids pP*_gene_-lacZ* and the pHT-P*_xyl_-vipR* (column “+vipR”) or the pHT-P*_xyl_* (column “−vipR”) were patched on LB medium containing X-gal (50 µg/mL) and xylose 20 mM and grown at 30°C for 24 h. The name of the gene whose promoter is fused to *lacZ* is indicated on the left. The picture is representative of at least three experiments.

The VipR transcriptional regulator of the Bt *kurstaki* HD1 strain positively controls its expression and the expression of the *vip3A* gene, located on the same plasmid (24). A conserved 32-bp sequence designated VipR box, whose integrity was critical for the expression of *vipR* and *vip3A,* was identified in their promoter region. This sequence was also identified in the promoter of five other transcription units on the pBMB299 plasmid: upstream of *cry2Ab*, *cry1I*, a three-gene operon containing *cry2Aa* and two genes annotated as N-acetylmuramoyl-l-alanine amidases (abbreviated as *ami*), with one *ami* gene being interrupted by a transposon (24). The consensus sequence of the VipR box was described using the IUPAC nucleotide code for the variable positions, and we used this sequence to search for other genes of the HD1 strain putatively regulated by VipR. No VipR box was found in the chromosome of the HD1 strain, but two additional occurrences of the VipR box were identified on plasmids. One sequence was located upstream from an *ami* gene on the pBMB65 (*ami65*), whereas the other was located upstream from an *ami* gene on the pBMB95 (*ami95*) (Fig. 1A; Fig. S1). Similarly to the other VipR boxes, the conserved sequences are located 17 nucleotides upstream from a putative Sig A −10 box. These results suggested that VipR was involved in regulating the expression of seven transcriptional units in the HD1 strain, all plasmid-encoded. In order to confirm that VipR was involved in the expression of these putative targets, we analyzed the transcriptional activity of DNA fragments located upstream from the coding sequence of *cry2Ab*, *cry1I*, the first gene of the *cry2Aa* operon, and the *ami95* gene located on the pBMB95. We used a heterologous host that does not harbor *vipR*, the Bt *kurstaki* HD73 Cry^−^ strain, which is genetically close to the HD1 strain. HD73 Cry^−^ is cured of the *cry1Ac*-carrying pHT73 plasmid (Table 1). Therefore, it does not produce any Cry toxin (26). Previous work has shown that an HD73 Cry^−^ strain that received the pBMB299 after a conjugative transfer from the HD1 strain was able to produce Vip3A, indicating that the genetic elements for the expression of the VipR-regulated *vip3A* gene and the regulator were functional in this strain despite the fact that it does not naturally contain the regulator (24).

**TABLE 1:** Plasmids used in this study

Name	Description
pBMB299	Native plasmid of the HD1 strain, carries the *vipR*, *vip3A*, *cry1IA*, *cry2Aa*, *cry2Ab*, and *cry1Aa* genes.
pBMB95	Native plasmid of the HD1 strain, carries the *cry1Ac* gene.
pHT73	Native plasmid of the HD73 strain, carries the *cry1Ac* gene.
pHT304.18Z	*E. coli/*Bt shuttle vector conferring resistance to ampicillin in *E. coli* and to erythromycin in Bt. Designed to allow transcriptional fusion with *lacZ* (27).
pP*_xyl_*	The pHT16.18K plasmid that contains the *xylR* repressor gene and the promoter of the *xylA* gene to allow gene expression upon xylose induction (28).
pP*_xyl_-vipR*	Plasmid allowing the expression of *vipR* under the xylose inducible promoter of *xylA* (24).
pHT-P*_cry1I_*	A 381 bp DNA fragment containing the *cry1I* promoter was PCR amplified using genomic DNA of the HD1 strain as the template and the primer pair Pcry1Ia-F/Pcry1Ia-R. The DNA fragment was ligated between the *Hind*III and *Bam*HI sites of the pHT304.18Z.
pHT-P*_cry2Aa_*	A 2,108 bp DNA fragment containing the promoter of the first gene of the *cry2Aa* operon was PCR amplified using genomic DNA of the HD1 strain as the template and the primer pair Pcry2Aa-F/Pcry2Aa-R. The DNA fragment was ligated between the *Sph*I and *Xba*I sites of the pHT304.18Z.
pHT-P*_cry2Ab_*	A 930 bp DNA fragment containing the promoter of the *cry2Ab* gene was PCR amplified using genomic DNA of the HD1 strain as the template and the primer pair Pcry2Ab-F/Pcry2Ab-R. The DNA fragment was ligated between the *Hind*III and *Bam*HI sites of the pHT304.18Z.
pHT-P*_ami95_*	A 199 bp DNA fragment containing the promoter of the *ami* gene was PCR amplified using genomic DNA of the HD1 strain as the template and the primer pair Pamidase-F/Pamidase-R. The DNA fragment was ligated between the *Hind*III and *Bam*HI sites of the pHT304.18Z.
pHT-P*_ami73_*	A 343 bp DNA fragment containing the promoter of the *ami* gene was PCR amplified using genomic DNA of the HD73 strain as the template and the primer pair Pamidase2-F/Pamidase-R. The DNA fragment was ligated between the *Hind*III and *Bam*HI sites of the pHT304.18Z.
pHT-P*_vipR_-vipR*	The pHT304.18Z that contains a 2,642 bp DNA fragment containing the promoter and the coding sequence of *vipR* between the *Bam*HI and *Hind*III sites (24).
pHT	The pHT304.18 *E. coli/*Bt shuttle vector conferring resistance to ampicillin in *E. coli* and to erythromycin in Bt (29).
pHT-*vipR*	The 2,642 bp DNA fragment that contains the promoter and the coding sequence of *vipR* between the *Bam*HI and *Hind*III sites of the pHT-P*_vipR_-vipR* was digested with the same enzymes and cloned in the pHT304.18.
pMAD	*E. coli/*gram-positive bacteria shuttle vector that contains a thermosensitive replication origin and conferring resistance to ampicillin in *E. coli* and to erythromycin in Bt. Designed to perform chromosomal genetic modification (30).
pMAD-∆*spo0A*	The upstream and downstream regions of the *spo0A* gene were amplified by PCR using chromosomal DNA from Bt strain HD73 Cry^−^ as template and primers spo0A-up-1/spo0A-up-2Bis and spo0A-dn-2/spo0A-dn-2Bis, respectively. The two fragments were fused using complementary sequences located in the primers, and the resulting DNA fragment was cloned between the *Bam*HI and *Xma*I sites of the pMAD vector.
pMADK	The DNA fragment containing the minimal thermosensitive replication origin of the pE194, the *BgaB* coding sequence, and the pBR222 origin of replication of the pMAD was amplified by PCR using the primer pair pMADopt-kana.FOR/pMADopt-kana.REV and the pMAD as the DNA template. The kanamycin resistance cassette was amplified using the kanaR.FOR/kanaR.REV primers and genomic DNA of the Bt407 *spo0A::apha3* strain (31) as DNA template. The two fragments were digested using *Bsa*I and ligated.
pMADK-∆*spo0A*	The 1,004 bp *Bam*HI-*Xma*I DNA fragment containing the upstream and downstream regions of the HD1 *spo0A* gene of the pMAD-∆*spo0A* plasmid was digested and cloned into the corresponding restriction sites of the pMADK.

The DNA fragments were fused to the promoterless β-galactosidase *lacZ* gene present on the pHT304.18Z, abbreviated as pHT-P*_gene_* (Table 1) (27). Each plasmid was introduced into the HD73 Cry^−^ strains carrying the pP*_xyl_-vipR* plasmid, allowing the expression of *vipR* under the control of the xylose-inducible promoter of *xylA*, or carrying the empty pP*_xyl_* plasmid (Table 1) (24). The HD73 Cry^−^ (pP*_xyl_-vipR*, pHT-P*_gene_*) and the HD73 Cry^−^ (pP*_xyl_*, pHT-P*_gene_*) strains (Table 2) were patched on LB plates containing X-gal and xylose. Blue colonies were observed after 24 h of growth for all the strains that carry the pP*_xyl-_vipR*, while the colonies were white for the pP_*xyl*_-carrying strains, indicating that the bacteria produced β-galactosidase activity only when the regulator was produced (Fig. 1B). A light blue coloration was observed for the HD73 Cry^−^ (pP*_xyl_*, pHT-P*_cry2Aa_*) strain after prolonged incubation, probably as a result of the activation of the SigE-dependent promoter included in the DNA cloned upstream of *lacZ* (Fig. S2B) (12). Quantification showed high levels of β-galactosidase activity for the pP*_xyl-_vipR*-harboring strains (29,126, 45,522, 87,188, and 33,605 U/mg protein for *cry1I*, *cry2Aa*, *cry2Ab*, and *ami95*, respectively) compared to those for strains carrying the empty vector (81, 1,183, 107, and 117 U/mg protein for *cry1I*, *cry2Aa*, *cry2Ab*, and *ami95*, respectively) (Fig. S2A). These results demonstrate that VipR positively regulates the expression of the HD1 genes that contain a VipR box in their promoter.

**TABLE 2:** Strains used in this study

Strain	Description	Antibiotic resistance
HD1	Bt *kurstaki* HD1 strain, belongs to the serotypes 3a–3c (32). It contains the pBMBLin15, pBM7635, pBMB8240, pBMB8513, pBMB14, pBMB46, pBMB64, pBMB65, pBMB74, pBMB95, pBMB299, and pBMB431.	
HD73 Cry^−^	Acrystalliferous Bt *kurstaki* HD73 strain, cured of the plasmid pHT73 carrying the *cry1Ac* gene (26). It contains the pHT7, pHT8_1, pHT8_2, pHT11, and pHT77. This strain is devoid of a *vipR* gene.	
HD73	Bt *kurstaki* HD73 strain that contains all the plasmids, belongs to the serotypes 3a–3c (32). It contains the pHT7, pHT8_1, pHT8_2, pHT11, pHT73, and pHT77. This strain is devoid of a *vipR* gene.	
HD73 Cry^−^ (pP*_xyl_*)	Strain HD73 Cry^−^ harboring the pP*_xyl_*, control strain used to measure the transcriptional activity of the VipR targeted genes.	Km^r^
HD73 Cry^−^ (pP*_xyl_*, pHT-P*_cry1I_*)	Strain HD73 Cry^−^ harboring the pP*_xyl_* and pHT-P*_cry1I_* plasmids, used to measure the transcriptional activity of the *cry1I* promoter.	Km^r^, Em^r^
HD73 Cry^−^ (pP*_xyl_*, pHT-P*_cry2Aa_*)	Strain HD73 Cry^−^ harboring the pP*_xyl_* and pHT-P*_cry2Aa_* plasmids, used to measure the transcriptional activity of the *cry2Aa* promoter.	Km^r^, Em^r^
HD73 Cry^−^ (pP*_xyl_*, pHT-P*_cry2Ab_*)	Strain HD73 Cry^−^ harboring the pP*_xyl_* and pHT-P*_cry2Ab_* plasmids, used to measure the transcriptional activity of the *cry2Ab* promoter.	Km^r^, Em^r^
HD73 Cry^−^ (pP*_xyl_*, pHT-P*_ami95_*)	Strain HD73 Cry^−^ harboring the pP*_xyl_* and pHT-P*_ami95_* plasmids, used to measure the transcriptional activity of the *ami95* promoter.	Km^r^, Em^r^
HD73 Cry^−^ (pP*_xyl_*, pHT-P*_ami73_*)	Strain HD73 Cry^−^ harboring the pP*_xyl_* and pHT-P*_ami73_*, used to measure the transcriptional activity of the *ami73* promoter.	Km^r^, Em^r^
HD73 Cry^−^ (pP*_xyl_-vipR*)	Strain HD73 Cry^−^ expressing *vipR* under the control of the xylose-inducible promoter of *xylA*, used to measure the transcriptional activity of the VipR targeted genes (24).	Km^r^
HD73 Cry^−^ (pP*_xyl_-vipR*, pHT-P*_cry1I_*)	Strain HD73 Cry^−^ harboring the pP*_xyl_-vipR* and pHT-P*_cry1I_* plasmids, used to measure the transcriptional activity of the *cry1I* promoter.	Km^r^, Em^r^
HD73 Cry^−^ (pP*_xyl_-vipR*, pHT-P*_cry2Aa_*)	Strain HD73 Cry^−^ harboring the pP*_xyl_-vipR* and pHT-P*_cry2Aa_* plasmids, used to measure the transcriptional activity of the *cry2Aa* promoter.	Km^r^, Em^r^
HD73 Cry^−^ (pP*_xyl_-vipR*, pHT-P_c*ry2Ab*_)	Strain HD73 Cry^−^ harboring the pP*_xyl_-vipR* and pHT-P*_cry2Ab_* plasmids, used to measure the transcriptional activity of the *cry2Ab* promoter.	Km^r^, Em^r^
HD73 Cry^−^ (pP*_xyl_-vipR*, pHT-P*_ami95_*)	Strain HD73 Cry^−^ harboring the pP*_xyl_-vipR* and pHT-P*_ami95_* plasmids, used to measure the transcriptional activity of the *ami95* promoter.	Km^r^, Em^r^
HD73 Cry^−^ (pP*_xyl_-vipR*, pHT-P*_ami73_*)	Strain HD73 Cry^−^ harboring the pP*_xyl_-vipR* and pHT-P*_ami73_* plasmids, used to measure the transcriptional activity of the *ami73* promoter.	Km^r^, Em^r^
HD73 (pHT)	Strain HD73 harboring the pHT plasmid, used as a control strain to study *cry1Ac* transcription and the production of pesticidal protein.	Em^r^
HD73 (pHT-*vipR*)	Strain HD73 harboring the pHT-*vipR* plasmid, constructed to study *cry1Ac* transcription and the production of pesticidal protein.	Em^r^
HD73 ∆*spo0A*	A *spo0A* mutant HD73 strain, defective for its ability to produce a spore.	
HD73 ∆*spo0A* (pHT)	Strain HD73 ∆*spo0A* harboring the pHT plasmid, constructed to study *cry1Ac* transcription and the production of pesticidal protein.	Em^r^
HD73 ∆*spo0A* (pHT-*vipR*)	Strain HD73 ∆*spo0A* harboring the pHT-*vipR* plasmid, constructed to study *cry1Ac* transcription and the production of pesticidal protein.	Em^r^
HD1 ∆*spo0A*	A *spo0A* mutant HD1 strain, defective for its ability to produce a spore. The strain contains the pBMBLin15, pBM7635, pBMB8240, pBMB8513, pBMB14, pBMB46, pBMB64, pBMB74, pBMB95, pBMB299, and pBMB431.	
HD1 (pHT-P*_ami95_*)	Strain HD1 harboring the pHT-P*_amy95_* plasmid, used to measure the transcriptional activity of the *ami95* promoter.	Em^r^
HD1 (pHT-P*_cry2Aa_*)	Strain HD1 harboring the pHT-P*_cry2Aa_* plasmid, used to measure the transcriptional activity of the *cry2Aa* promoter.	Em^r^
HD1 (pHT-P*_cry2Ab_*)	Strain HD1 harboring the pHT-P*_cry2Ab_* plasmid, used to measure the transcriptional activity of the *cry2Ab* promoter.	Em^r^
HD1 ∆*spo0A* (pHT-P*_ami95_*)	Strain HD1 harboring the pHT-P*_amy95_* plasmid, used to measure the transcriptional activity of the *ami95* promoter.	Em^r^
HD1 ∆*spo0A* (pHT-P*_cry2Aa_*)	Strain HD1 harboring the pHT-P*_cry2Aa_* plasmid, used to measure the transcriptional activity of the *cry2Aa* promoter.	Em^r^
HD1 ∆*spo0A* (pHT-P*_cry2Ab_*)	Strain HD1 harboring the pHT-P*_cry2Ab_* plasmid, used to measure the transcriptional activity of the *cry2Ab* promoter.	Em^r^

Interestingly, when we searched for a VipR box in the naturally *vipR*-less HD73 strain, one occurrence was found upstream from an *ami* gene located on the pHT73 plasmid (*ami73*). Similarly to the HD1 strain, no VipR box was identified in the chromosome of the HD73 strain. To determine whether the expression of this gene depends on VipR, the pHT-P*_ami73_* plasmid (Table 1) was introduced into the HD73 Cry^−^ (pP*_xyl_*) and HD73 Cry^−^ (pP*_xyl_-vipR*) strains (Table 2). HD73 Cry^−^ (pP*_xyl_*, pHT-P*_ami73_*) colonies were white when plated on LB plates containing X-gal and xylose (Fig. 1B). In contrast, the HD73 Cry^−^ (pP*_xyl_-vipR*, pHT-P*_ami73_*) colonies were blue when *vipR* was expressed, and a high level of β-galactosidase activity was measured (31,199 U/mg protein at 24 h, compared to 94 U/mg protein in the absence of *vipR*) (Fig. S2A), indicating that the *ami73* gene was expressed in the HD73 strain only after the introduction of the *vipR* gene.

### The *ami73* and *cry1Ac* genes belong to the same transcription unit

**Fig 2:**
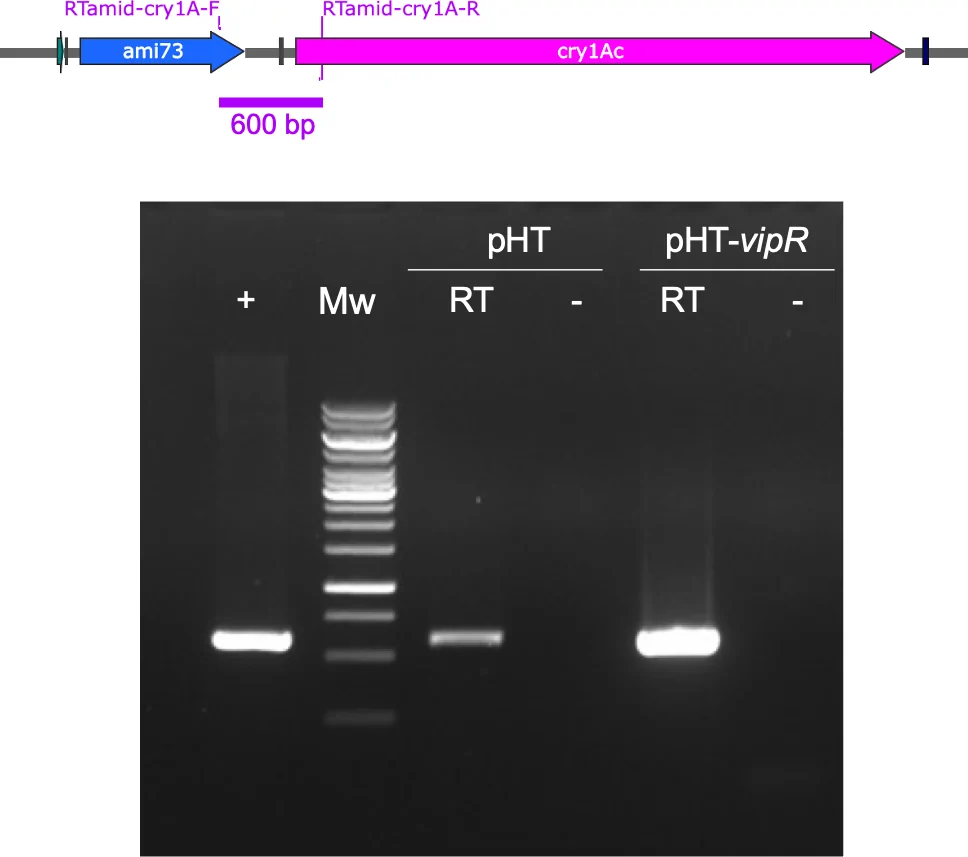
Analysis of the *ami73-cry1Ac* operon in the HD73. A schematic of the *ami73-cry1Ac* locus shows the localization of the primers used for the RT-PCR experiment. Agarose gel electrophoresis analysis of the production of a 600 bp PCR product obtained with the RTamid-cry1A-F and RTamid-cry1A-R primer pair and HD73 genomic DNA as the DNA template (lanes +), or HD73 (pHT) and HD73 (pHT-vipR) RNA treated (lanes RT), or not (lanes −), with the reverse transcriptase. RNA was collected 2 h after the onset of stationary phase from bacteria grown in LB medium at 30°C. Mw, GeneRuler 1 kb DNA molecular weight ladder. The picture is representative of two experiments.

A comparison of all the HD1 and HD73 *ami* loci shows that the promoter and the coding sequence of the *ami73* are identical to those of the *ami65* gene and to the promoter of the transposon-interrupted ami299* gene on the pBMB299. A few point mutations and a small insertion differentiate the *ami299* and *ami95* sequences (Fig. S1). Besides the conservation of the *ami* sequences, we noticed that the *ami65*, *ami95*, *ami299*, and *ami73* genes were located upstream from a *cry1A* gene (Table S1). In the strain HD73, the *ami73* and *cry1Ac* genes are separated by a 289-bp intergenic region that lacks a putative rho-independent transcription terminator (ARNold web server). This suggested that the two genes could belong to the same transcription unit. To test this hypothesis, we performed an RT-PCR experiment with the HD73 strain carrying the pHT-*vipR*, allowing the expression of the regulator under its own promoter, and the HD73 (pHT) carrying the empty pHT304.18 (Tables 1 and 2). RNA was collected at T2 from bacteria grown in LB when VipR was shown to be active (24). The primer pair RTamid-cry1A-F and RTamid-cry1A-R (Table 3) was used to detect the transcription of the putative operon (Fig. 2; Fig. S3). An amplicon was produced both in the presence and the absence of *vipR*, indicating co-transcription of the genes, although at a low level in the absence of *vipR*. The higher intensity of the PCR product produced with RNA extracted from the *vipR*-carrying strain suggests that VipR increases the transcription of the operon. This result also suggested that VipR can activate the expression of *cry1Ac* early in the stationary phase and independently of the sporulation promoters located in the intergenic region upstream from the *cry1Ac* coding sequence. Indeed, SigE and SigK are not yet activated at this point when bacteria are grown in LB (16). The faint VipR-independent transcription of the *ami73-cry1Ac* operon detected by RT-PCR (Fig. 2; Fig. S3), but not in the β-galactosidase assay (Fig. 1B), is presumably due to a weak promoter located upstream of the DNA fragment cloned in the pHT-P*_ami73_* construct used in the transcription assay.

**TABLE 3:** Primers used in this study^*a*^

Name	Sequence (5′−3′)	Restriction site
Pcry1Ia-F	CCC**AAGCTT**GTGATGTCCGTTTTACTATGTTATTTTCTAG	HindIII
Pcry1Ia-R	CG**GGATCC**ACTTATACTATTATTTAATTTTCAGATATAAT	BamHI
Pcry2Aa-F	A**GCATGC**AAAGATTCCAACAAGTTTATTAGAGC	SphI
Pcry2Aa-R	A**TCTAGA**CTTCTACTTTCATTTATGGGTTTC	XbaI
Pcry2Ab-F	CCC**AAGCTT**AGAATGAAGCAATATGACTTCTAACC	HindIII
Pcry2Ab-R	AA**GGATCC**TTAAATATTAAGTAATATATTAAAATTAAAGATAC	BamHI
Pamidase-F	CCC**AAGCTT**ATCACGAAAAGAAGTTTTTAAGAACCATTCC	HindIII
Pamidase-R	CG**GGATCC**TATCAGATTATTGGATATGATTTCAATCTTTC	BamHI
Pamidase2-F	CCC**AAGCTT**TGTAGAAACTTTGTAATTTGTATGAATTGAG	HindIII
RTamid-cry1A-F	GGTCTATGGTTCTGCTAATTGG	
RTamid-cry1A-R	GAACAAATTCACTCAAAAGAAATTGC	
spo0A-up-1	CG**GGATCC**TGAAGTTGTTAGTACAGTGCAGC	BamHI
spo0A-up-2Bis	AAATCCCTTACTACTTTTACTCTCAGTGTATCCTTCTAGGCTACATGA	
spo0A-dn-2	TCCC**CCCGGG**GCTGTAATGACTTCGCTTTCAGT	XmaI
spo0A-dn-2Bis	TCATGTAGCCTAGAAGGATACACTGAGAGTAAAAGTAGTAAGGGATTT	
pMADopt-kana.FOR	TAT**GGTCTC**ACGATGGGGCCAGATGGTAA	BsaI
pMADopt.REV	TAT**GGTCTC**ATCGTGCTGCGAGGGC	BsaI
kanaR.FOR	TAT**GGTCTC**AACGAGATAAACCCAGCGAACCATTTGAGG	BsaI
KanaR.REV	TAT**GGTCTC**AATCGATACAAATTCCTCGTAGGCG	BsaI

^*a*^ Restriction enzyme sites are indicated by bold nucleotides.

### VipR controls the early production of Cry1Ac

**Fig 3:**
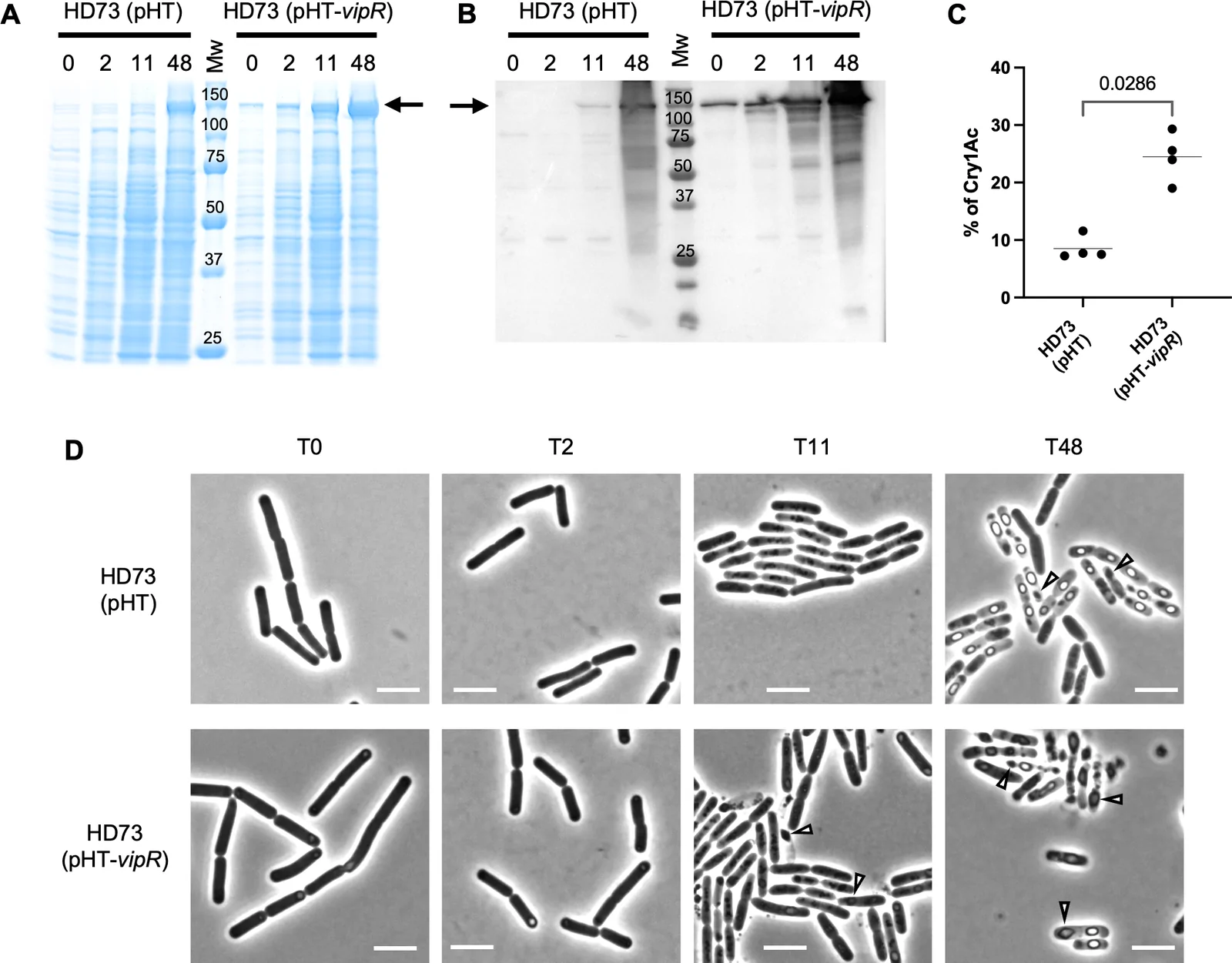
Cry1Ac production in the presence of VipR in the HD73 strain. (**A**) SDS-PAGE analysis of total protein extracts of the HD73 (pHT) and HD73 (pHT-*vipR*) strains. Bacteria were grown in LB medium containing 5 µg/mL erythromycin at 30°C and collected at the indicated time (in hours). Proteins were prepared as described in Materials and Methods. Three microliters of sample was loaded in each well. The arrow points to a 133 kDa-migrating protein band that corresponds to Cry1Ac. T0 corresponds to the entry into the stationary phase. Mw, protein molecular weight marker. The picture is representative of four independent experiments. (**B**) Western blot analysis of Cry1Ac production by the same strains as in panel A. A 0.3 µL protein sample was loaded in each well, transferred to PVDF membrane, and probed with an antiserum raised against the Bt *aizawai* 7.29 crystal proteins. The picture is representative of two independent experiments. (**C**) Proportion of Cry1Ac in HD73 (pHT) and HD73 (pHT-*vipR*) strains. The proportion of Cry1Ac in SDS-PAGE gel (as in panel A) was calculated by densitometry and expressed as the percentage of Cry1Ac in a protein extract at 48 h. The data are the result of four independent experiments, and the bar represents the mean, *n* = 4. Result of a Mann-Whitney statistical test is indicated in gray with the *P* value. (**D**) Time course microscopy observations of the HD73 (pHT) and HD73 (pHT-*vipR*) strains grown in LB medium at 30°C for 48 h. Arrowheads point to typical Cry1Ac bipyramidal crystals. Scale bar corresponds to 5 µm.

To determine whether the VipR-dependent *cry1Ac* expression could lead to the production of the insecticidal toxin, we decided to use the HD73 (pHT-*vipR*) strain expressing *vipR* cloned under the control of its own promoter in the pHT304.18 plasmid in order to work with a minimal VipR-target system. The low-copy-number (four per chromosome) of the pHT304 replicon (29) should not cause a significant change compared to the copy number of the pBMB299 plasmid, estimated at two per chromosome (Table S2). The protein profile of the strains HD73 (pHT-*vipR*) and HD73 (pHT) was analyzed (Table 2). Bacteria were grown in LB medium, and samples were collected at different time points during the stationary phase, starting from T0, which defines the transition between the exponential and stationary phases. The SDS-PAGE and Western blot analyses indicated that in the presence of *vipR*, a band corresponding to Cry1Ac was detected from T0 and accumulated over time (Fig. 3A and B). In the absence of *vipR*, Cry1Ac was detected in HD73 (pHT) samples only during the late stationary phase, at T11 and later, due to SigE- and SigK-dependent *cry1Ac* expression (16). These results indicated that VipR allows the production of the crystal protein from the beginning of the stationary phase of growth. The amount of Cry1Ac protein produced by the two strains was compared by densitometry (Fig. 3C). The quantification of the proportion of the toxin in the 48-h samples showed that this protein represented, on average, 8.5% of the protein content for the HD73 (pHT) strain. In contrast, this proportion increased to 24.5% for the HD73 (pHT-*vipR*) strain. In agreement with these quantifications, microscopy analysis showed that the *vipR*-carrying strain produced crystals that appeared earlier and seemed bigger than in the *vipR*-less strain (Fig. 3D).

### VipR allows Cry toxin production in a sporulation mutant strain

**Fig 4:**
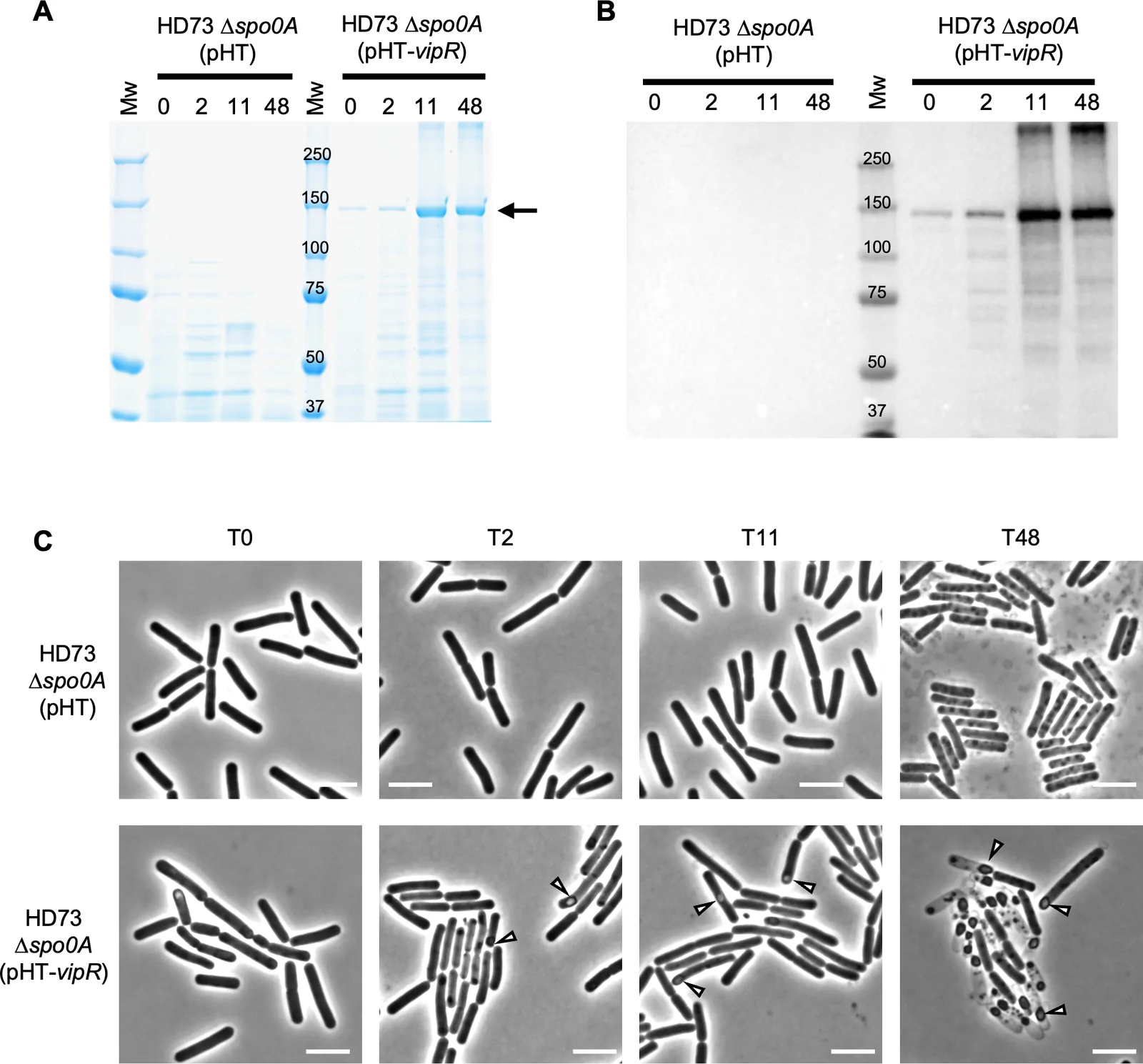
Cry1Ac production in the presence of VipR in the HD73 Δ*spo0A* strain. (**A**) SDS-PAGE analysis of total protein extracts of the HD73 Δ*spo0A* (pHT) and HD73 Δ*spo0A* (pHT-*vipR*) strains. Bacteria were grown in LB medium containing 5 µg/mL erythromycin at 30°C and collected at the indicated time points (in hours). The arrow points to a 133 kDa-migrating protein band that corresponds to Cry1Ac. Proteins were prepared as described in Materials and Methods. Three microliters of sample was loaded in each well. T0 corresponds to the entry into the stationary phase. Mw, protein molecular weight marker. The picture is representative of four independent experiments. (**B**) Western blot analysis of Cry1Ac production by the same strains as in panel A. A 0.3 µL protein sample was loaded in each well, transferred to PVDF membrane, and probed with an antiserum raised against the Bt *aizawai* 7.29 crystal proteins. The picture is representative of three independent experiments. (**C**) Time course microscopy observations of the HD73 Δ*spo0A* (pHT) and HD73 Δ*spo0A* (pHT-*vipR*) strains grown in LB medium at 30°C for 48 h. Arrowheads point to typical Cry1Ac crystalline inclusions. Scale bar corresponds to 5 µm.

To assess if Cry1Ac could be produced in a sporulation mutant, we constructed the HD73 ∆*spo0A* strain in which the sporulation process is blocked in its early steps and SigE and SigK are not produced (Table 2) (33, 34), as in *Bacillus subtilis* (35). The protein profile of the strains HD73 ∆*spo0A* (pHT) and HD73 ∆*spo0A* (pHT-*vipR*) (Table 2) was compared when bacteria were grown in LB medium (Fig. 4A and B). The HD73 ∆*spo0A* (pHT) did not produce Cry1Ac. In sharp contrast, in the HD73 ∆*spo0A* (pHT-*vipR*) samples, a faint production of Cry1Ac was detected from T0 and accumulated over time. Microscopy pictures of the samples showed that the HD73 ∆*spo0A* (pHT) strain did not produce spores or crystals (Fig. 4C). In contrast, crystal-like inclusions were observed in some HD73 ∆*spo0A* (pHT-*vipR*) bacteria in the samples collected at T0 and in more cells over time. At 48 h, large crystals could be observed in the cells. However, the typical bi-pyramidal structure of the crystals produced by the sporulation-deficient cells seemed less conserved than in sporulating bacteria (Fig. 3D and 4C). These results demonstrate that VipR activates the expression and production of the Cry1Ac toxin independently of the sporulation, presumably from the VipR-dependent promoter located upstream from the *ami* gene. In agreement with the *vipR* transcription profile in a ∆*spo0A* strain (24), the *vipR*-dependent Cry1Ac production occurs from the onset of the stationary phase.

### VipR-dependent Cry toxins production in a sporulation mutant HD1 strain

As the work presented above was carried out with our model strain HD73, we wanted to confirm that the VipR-dependent Cry toxin production in ∆*spo0A* bacteria also occurred in the HD1 strain that naturally contains a *vipR* gene. In this strain, VipR might not only control the expression of the three *cry1A* genes but also of *cry1I*, *cry2Ab*, and the operon that contains the *cry2Aa* gene (Table S1). Having failed to construct a sporulation mutant in the HD1 strain with the plasmid pMAD, probably because of the selection on erythromycin, as no transformants were obtained, we designed the pMADK plasmid harboring a gene conferring resistance to kanamycin instead of erythromycin (see Table 1, Fig. S4, and Materials and Methods for details). Using this vector, we successfully constructed the HD1 ∆*spo0A* strain (Table 2). While verifying the mutant strain, we noticed that the *cry1Ab* carrying pBMB65 plasmid had been lost, probably during the culture steps at 40°C to block replication of the thermosensitive pMADK after the recombination events. The HD1 ∆*spo0A* is, therefore, also a Cry1Ab^−^ strain.

**Fig 5:**
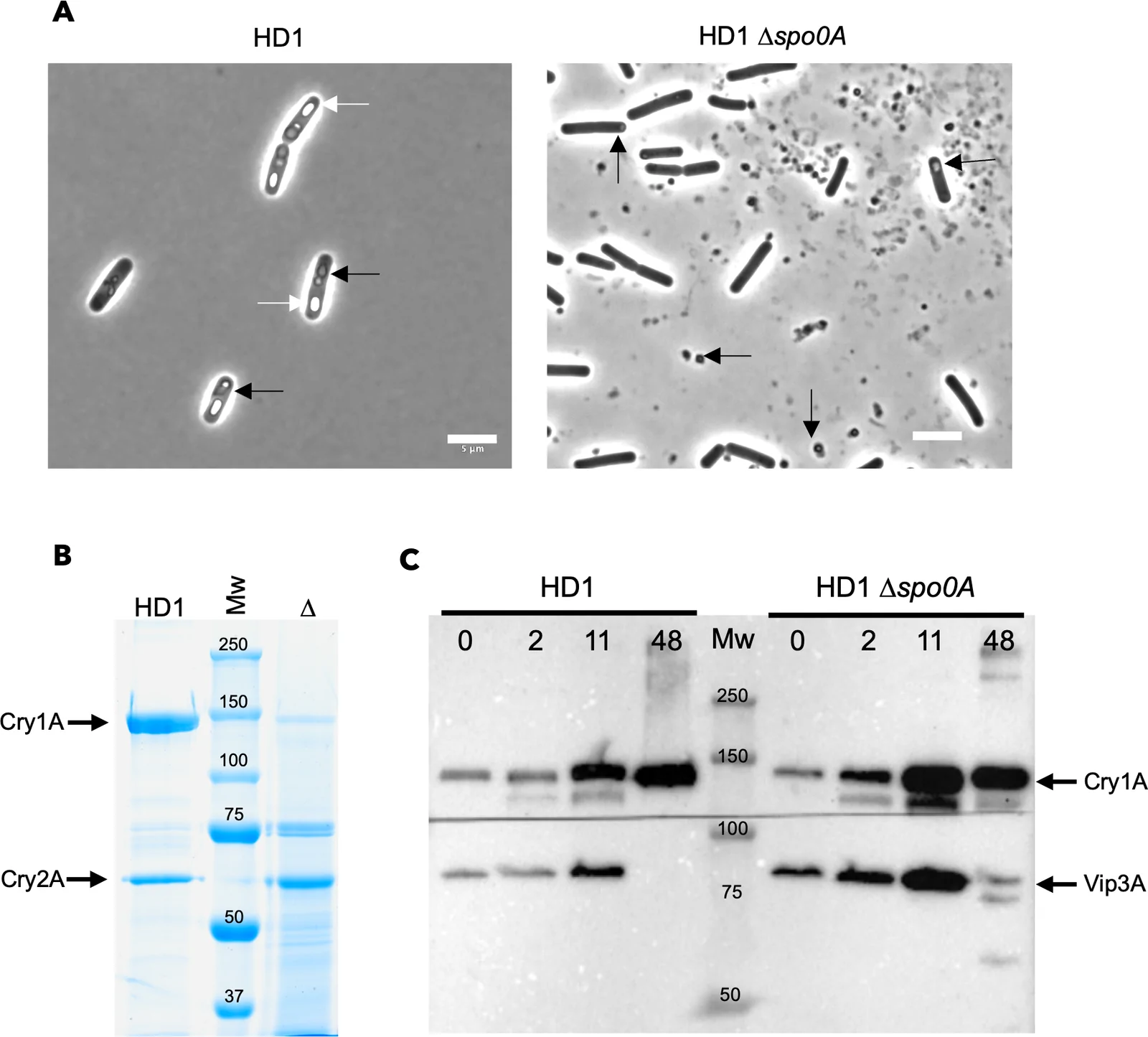
Production of insecticidal proteins by a sporulation-deficient HD1 strain. (**A**) Microscopy observations of the HD1 wt and Δspo0A strains grown on LB plates at 30°C for 48 h. Black arrows point to crystalline inclusions, liberated or not. White arrows point to endospores. Scale bar corresponds to 5 µm. Microscopy experiments were repeated twice. (**B**) SDS-PAGE analysis of 1.5 µg and 12 µg of a total protein extract of the HD1 (lane HD1) and HD1 Δ*spo0A* (lane HD1 Δ) strains, respectively. Bacteria were grown on LB medium plates at 30°C for 2 days, followed by an incubation at room temperature for 6 days. Arrows point to the 133 kDa Cry1A proteins and the 70-kDa Cry2A proteins. Mw, protein molecular weight marker. Representative picture of two experiments. (**C**) Western blot analysis of Vip3A and Cry1Ac production by the HD1 wt and HD1 ∆*spo0A* strains over time. Identical volumes of total extracts were loaded in each well except for the HD1 T48 sample, for which a 20× dilution of the sample was used. Proteins were transferred to a PVDF membrane. The membrane was cut, and the bottom part was probed with a monoclonal anti-Vip3Aa antibody, and the top part was probed with an antiserum that recognizes Cry1A proteins from the Bt *aizawai* 7.29 strain.

To determine the expression profile of the *cry1 and cry2* genes in the HD1 strain, the pHT-P*_ami95_*, pHT-P*_cry2Aa_*, and pHT-P*_cry2Ab_* (Table 1) were introduced in both wt and ∆*spo0A* strains (Table 2). Transcriptional analysis of *ami95*, *cry2Aa*, and *cry2Ab* promoters showed the activation of these VipR-dependent promoters from the beginning and during the stationary phase in both the wt and *spo0A* mutant strains (Fig. S5). Microscopy analyses were then conducted to evaluate the production of crystals in the HD1 and HD1 ∆*spo0A* strains (Fig. 5A). The HD1 ∆*spo0A* bacteria did not produce a spore as expected. However, we observed the presence of small inclusions resembling square crystals inside some cells, and structures that looked like bipyramidal crystals that were mostly liberated in the growth medium. Large crystals of different shapes were produced as well as spores by the HD1 wt cells (Fig. 5A). The analysis of the protein profile of the two strains, grown on plates until late stationary phase, by SDS-PAGE showed that proteins with a molecular weight corresponding to Cry1A and Cry2A (130 and 60 kDa, respectively) were present in both the wt and ∆*spo0A* samples (Fig. 5B), indicating that at least one of the Cry1A and Cry2A toxins was produced by the HD1 ∆*spo0A* strain. The identity of the proteins migrating at 130 and 60 kDa was confirmed by mass spectrometry (Table 4). We also analyzed the two intense 75 kDa protein bands and found a large production of InhA1, InhA2, and InhA3 by the sporulation mutant. A complementary shotgun proteome analysis of the same HD1 ∆*spo0A* sample confirmed the production of Cry1Aa, Cry1Ac, Cry1Ia, Cry2Aa, Cry2Ab, and Vip3Aa (Table 4). We also conducted an analysis of selected insecticidal protein production over time in both genetic backgrounds and we observed the production of Cry1A and Vip3A proteins from T0 (Fig. 5C). These results demonstrate that early and significant production of insecticidal proteins occurs independently of the sporulation in a strain that naturally carries a *vipR* gene and toxin genes that contain a VipR box in their promoter region.

**TABLE 4:** The most abundant proteins identified in the by LC-MS/MS in the HD1 Δ*spo0A* sample^*a*^

Locus tag	Protein	Log(*E* value)	Coverage	Spectra	Specific spectra	Unique peptides	Theoretical number of peptides	PAI
BTK_RS04085	InhA2	−494.16	48.69%	221	205	116	33	3.52
BTK_RS08025	InhA1	−473.60	41.21%	213	200	90	30	3.00
BTK_RS15040	InhA3	−327.05	37.23%	119	110	67	34	1.97
	Lysozyme C	−481.56	89.12%	427	427	106	10	10.60
BTK_RS32325	Cry2Ab	−335.79	50.55%	137	99	74	25	2.96
BTK_RS32270	Cry2Ab	−364.81	49.76%	135	97	72	26	2.77
BTK_RS13765	Non-ribosomal peptide synthetase	−190.55	10.26%	63	53	47	214	0.22
BTK_RS13760	Non-ribosomal peptide synthetase	−106.66	12.01%	37	30	25	86	0.29
BTK_RS13755	Non-ribosomal peptide synthetase	−120.87	15.28%	32	25	24	60	0.40
BTK_RS32295	Cry1Aa	−241.67	25.09%	106	44	47	38	1.24
BTK_RS31195	Cry1Ac	−213.94	25.57%	99	40	46	41	1.12
BTK_RS32300	Cry1Ia	−148.08	36.16%	57	54	37	26	1.42
BTK_RS32255	Vip3Aa	−217.23	34.85%	68	68	49	32	1.53

^*a*^ Coverage, the percentage of the protein sequence covered by the peptides identified; ppectra, the number of spectra that identified the protein; ppecific spectra, the number of specific spectra only assigned to this protein; unique peptides, number of unique peptide sequences attributed to this protein, used to compute the PAI; theoretical number of tryptic peptides, used in the PAI computation; and protein abundance index (PAI), the number of peptides identified divided by the number of theoretically observable tryptic peptides.

### *In silico* analysis of VipR-dependent gene occurrences within the Bt species

While this experimental work has been performed on two Bt *kurstaki* strains, a previous analysis of the presence of the *vipR* gene in Bt strains showed that the regulator gene is present in strains belonging to the *galleriae*, *aizawai*, *chinensis*, *alesti*, *thuringiensis*,* kurstaki*, *dendrolimus*, and *morrisoni* serovars (24).

**Fig 6:**
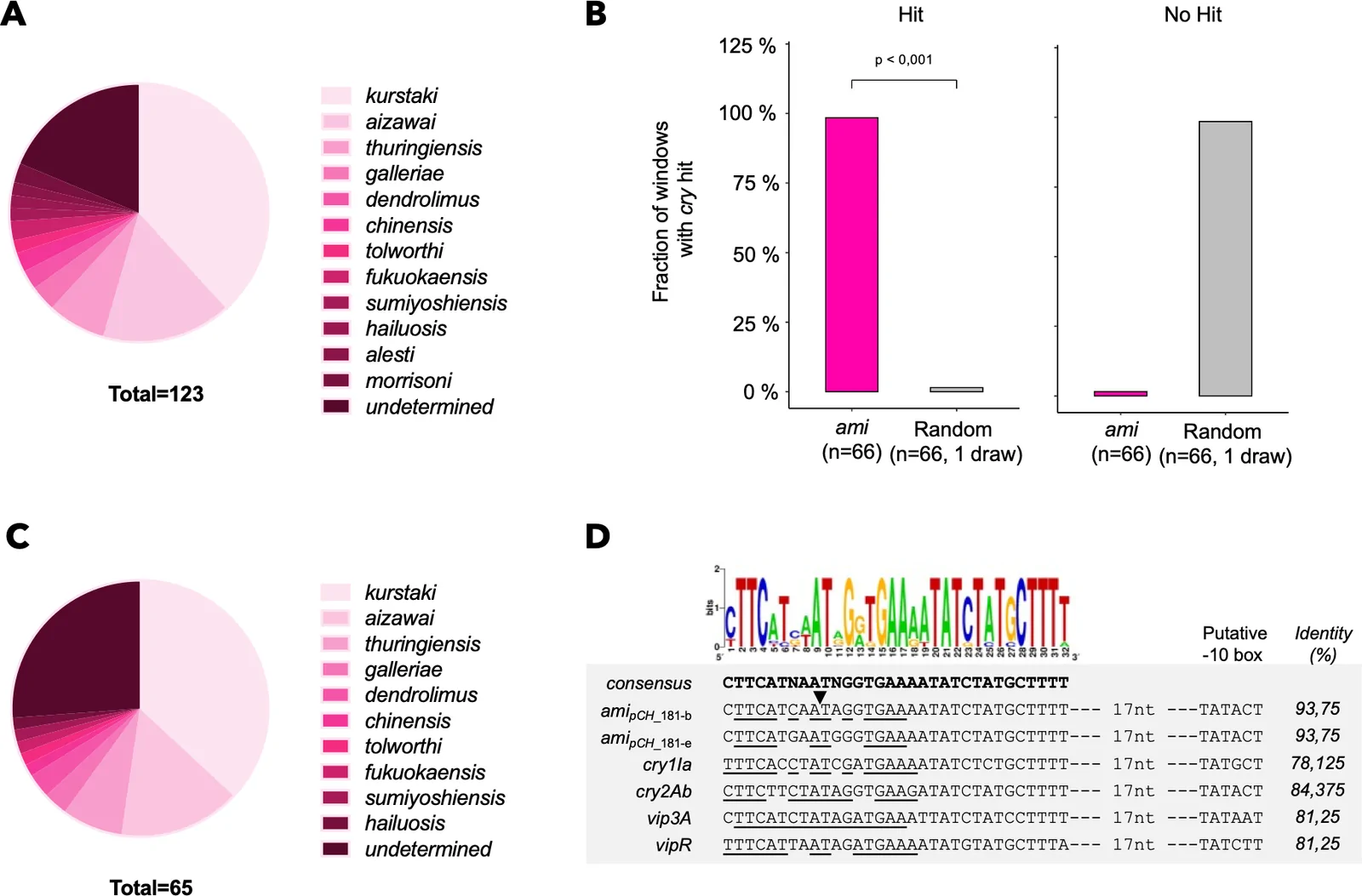
Genome-wide analyses of the VipR-regulated *cry* gene expression. (**A**) Distribution of the 123 *cry* genes containing a VipR box in their promoter according to their serovar. Each color corresponds to a Bt serovar. (**B**) *ami* loci are enriched in *cry* genes. *ami* gene homologs were identified by blast in Bt genomes, and the presence of a *cry* gene in a 10-kb window centered around each *ami* gene was searched by running a tblastn using the sequence of all Cry holotypes (*ami* column). Identification of a *cry* gene in the 10-kb DNA window provides a hit. Absence of a *cry* gene is considered as a no hit. The same analysis was done on the Bt genomes divided into 10-kb DNA fragments (Random column). A permutation test (10k random draws) was performed and concluded to a statistically significant enrichment in *cry* genes of the *ami* loci (*P* < 0.001). (**C**) Distribution of the 65 *ami-cry* loci identified in Bt genomes according to their serovar. Each color corresponds to a Bt serovar. (**D**) Alignment of the VipR boxes present in the genome of the Bt *aizawai* ABTS-1857 strain. The name of the gene that contains the conserved sequence in its promoter is indicated on the left. The distance between the putative −10 box and the last nucleotide of the box is indicated. The VipR box consensus, composed of base frequency with a threshold >50% in the 10 sequences used in the alignment, and the sequence logo constructed with the Bt *kurstaki* HD1 and HD73 sequences are shown on top of the alignment. Underlined nucleotides correspond to the palindrome (center is indicated with an arrowhead). Percentage of identity compared to the consensus sequence is indicated on the right.

Bioinformatics analysis was conducted in order to determine the extent of the presence of a VipR box in the promoter regions of *cry* genes. *cry* DNA sequences were identified in the NCBI Bt genome database using a tblastn search with the Cry holotype sequences collected from the Bacterial Pesticidal Protein Resource Center (www.bpprc-db.org) in order to constitute a *cry* gene database. For each *cry* gene identified, the upstream 2,100 bp long DNA sequence was screened for the presence of a VipR box sequence. Our stringent analysis gave a total of 123 hits and allowed us to extend the catalog of the putative VipR-regulated Cry targets to Cry1Ae, Cry1Ah, Cry1Ea, Cry1Fb, Cry1Ia, Cry1Ja, Cry2Ad, Cry2Ah, Cry9Da, and Cry9Ee genes, in addition to Cry1Aa, Cry1Ab, Cry1Ac, Cry1Ia, Cry2Aa, and Cry2Ab (Fig. 6A; Table S3). The genes encoding these Cry proteins were identified in strains belonging mostly to the *kurstaki* and *aizawai* serovars but also *thuringiensis*, *galleria*, *dendrolimus*, *chinensis*, *tolworthi*, *fukuokaensis*, *sumiyoshiensis*, *hailuosis*, *alesti,* and *morrisoni,* as well as in strains lacking information about the serotype but containing insecticidal genes with predicted anti-lepidopteran activity as well.

We then sought to determine if the *ami-cry1A* genetic organization identified in the *kurstaki* strains was present in other Bt genomes. Sixty-six homologs of *ami_299_* were identified by blastn in the Bt genome database. Search for insecticidal protein genes in 10-kb DNA windows centered around the *ami* gene was performed using tblastn with the Cry holotype sequences. The result of this analysis showed an enrichment in *cry* genes in the *ami* genomic neighborhood compared to the same analysis done on sequences corresponding to 10-kb random genomic DNA fragments (Fig. 6B). Analysis of the best hit *cry* sequences associated with the 66 *ami* loci identified 65 *cry* genes. One locus did not give a positive hit because an incomplete *cry* gene was present downstream of the *ami* gene and did not pass our filters. Analysis of the *cry* gene sequences indicated that 62 hits were *cry1A* genes, one hit was a *cry1E* DNA sequence, and two hits were *cry9E* genes (Table S4). The latter were identified in strains Bt serovar *fukuokaensis* 4AP1 and Bt serovar *sumyoshiensis* 4AO1. The other *amy-cry* loci were mostly present in strains belonging to the *kurstaki*, *aizawai*, and *thuringiensis* serovars (Fig. 6C; Table S4). As the serotype of strains is not systematically determined, the *ami-cry* genetic organization was also found in strains with undefined serovar. These results show that the genetic organization of the *ami-cry1A* described for Bt *kurstaki* (Fig. 2) is present in various serovars. However, the cotranscription of these genes has yet to be demonstrated. Our data also suggest that coexpression of the *ami* gene with *cry* protein genes other than *cry1* may occur.

Interestingly, some strains carrying *vipR* and VipR boxes may also harbor other insecticidal protein genes not preceded by a VipR box but where typical *sigE* and *sigK* promoters are present. An example of such a strain is the Bt *aizawai* ABTS-1857 (36), where the pCH181-b harbors a *vipR* gene and four insecticidal protein genes, *vip3Aa, cry2Ab*, *cry1Ia*, and an *ami-cry1Aa* operon with a VipR box in their promoter (Fig. 6D; Table S1), as well as *cry1Ca* and *cry1Da* genes that do not present a VipR box, but harbor typical SigE and SigK recognition sequences in their promoters (Table S1; Fig. S6). This strain also carries another *ami-cry1Ab* operon on the pCH181-e consistent with a VipR-controlled transcription unit (Fig. 6D) as well as a *cry9Ea* gene located on the pCH181-a plasmid and whose promoter does not contain a VipR box (Table S1; Fig. S6). In contrast to the *cry9Ee* genes from Bt *fukuokaensis* 4AP1 and Bt *sumyoshiensis* 4AO1 strains, no *ami* gene is present upstream of the Bt *aizawai* ABTS-1857 *cry9Ea* coding sequence. Similarly to the *kurstaki* strains, no VipR box was found in the chromosome of the ABTS-1857 strain.

## DISCUSSION

For decades, Cry toxin production has been considered to be intimately linked to sporulation, and rightly so. The sporulation sigma factors-dependent *cry* gene expression is a key element enabling the concomitant formation of the spore and the crystal in the mother-cell compartment, with the *cry1* and *cry2* genes being typical examples (9, 12, 13). This finely tuned gene regulation could be seen as a strategy to produce the perfect set of weapons to successfully achieve the infection of a susceptible insect and the completion of the bacteria's life cycle (37). However, the sporulation-independent expression of the *cry3A* gene (17) and identifying the transcription factor CpcR in Bt strain LM1212 (20), the first example of a regulator activating *cry* gene expression in non-sporulating cells, have shed new light on the regulation of these toxin genes. VipR, a transcriptional regulator that belongs to the PCVR family, was previously identified as the transcriptional regulator that controls the expression of the Vip3A insecticidal toxin gene (24). With this present work, we demonstrate that in the HD1 strain, VipR is a key player in the expression of a subset of insecticidal protein genes, including *cry1Aa*, *cry1Ab*, *cry1Ac*, *cry2Aa*, and *cry2Ab,* whose transcription was previously thought to be exclusively sporulation-dependent (Fig. 1B) (14), as well as the expression of the insecticidal proteins liberated in the growth medium, Vip3A and Cry1I. An unexpected result of our work is related to the expression of the *cry2Ab* gene, considered to date as a cryptic gene (10, 38–40) even though peptides that can be attributed specifically to Cry2Ab have been identified in samples of the DiPel DF (ABTS-351) and XenTari GD (ABTS-1857) commercial products (41). While this protein can be efficiently produced as a P*_cry3A_-cry2Ab* promoter-gene fusion (39), two studies have unsuccessfully addressed the natural *cry2Ab* regulation. A first study searched for promoter sequences resembling those preceding other *cry1A* or *cry2Aa* genes but only within the 300 bp upstream from the *cry2Ab* coding sequence (10). No such promoter was identified, which is in agreement with the fact that the VipR-dependent promoter is located more than 700 bp upstream from the *cry2Ab* ORF. The second study used *E. coli* and an HD73 crystal-negative mutant strain as alternative Cry2Ab producers (39). While the whole intergenic region upstream from *cry2Ab*, in which the VipR box is located, was comprised in the DNA construct introduced in these heterologous hosts, Cry2Ab production was not detected. These results are in line with the fact that the *vipR* gene is absent in the genome of these hosts, whereas it is present in strains DiPel DF (ABTS-351) and XenTari GD (ABTS-1857).

An analysis of the VipR-regulated *ami* genes loci drew our attention to an unexpected role of VipR in the expression of the downstream *cry1A* gene, independently of the SigE- and SigK-dependent promoters located in the intergenic region between the *ami* and *cry1A* ORFs (Fig. S1). To demonstrate this, we confirmed that the *ami* and the *cry1Ac* genes, previously described in a genomic study as being the minimal *cry1* genetic unit (42), belonged to a same transcription unit (Fig. 2). We also demonstrated that *vipR* enables the expression and production of the endogenous Cry1Ac from the VipR-dependent *ami* promoter (Fig. 3A and B, 4A and B, and 5C) at the beginning of the stationary phase, hours before the activation of SigE, which only occurred approximately 11 h after the onset of stationary phase when bacteria are grown in LB medium (16). A faint transcription of *cry1Ac* originating from the intergenic region upstream of the *cry1A* open reading frame, and independent from the sporulation promoters, was also described in strain HD73 (16). Spo0A seems to positively regulate this low expression during the stationary phase, but the cause of this activation was not identified. While we also used an HD73 strain in our experiments, we did not observe an early expression and production of Cry1Ac that could originate from the DNA region directly upstream from the *cry1Ac* gene (Fig. 3A and B), and we could not find a VipR box in this DNA sequence. In line with this idea, the detection of Cry1Ac from T0 was only observed when a VipR expression cassette was introduced in the strain, thus, with a transcriptional activation from the *ami73* promoter. This *ami-cry1A* genetic organization was not only found in strains belonging to the *kurstaki* serovar but also in *aizawai*, *thuringiensis*, *galleriae*, *chinensis*, *tolworthi*, *dendrolimus*, and *hailuosis* strains. Interestingly, our genome-wide analysis of *ami* homologs identified the occurrence of *ami-cry9E* loci in Bt ser*ovar fukuokaensis* 4AP1 and Bt *serovar sumyoshiensis* 4AO1 strains (Fig. 6B and C; Table S4). As no terminator was identified between the two coding sequences, their organization as an operon is probable, suggesting a production from the VipR-regulated *ami* promoter in a time frame similar to the *vip3A* gene expression, also present in these strains. In these strains, a synergistic insecticidal toxicity between Vip3A and Cry9 towards *Chilo suppressalis* was demonstrated, suggesting a coevolution of these genes (43, 44). A shared mechanism of gene expression could synchronize the production of the insecticidal proteins required for a synergistic effect. Identification of the VipR boxes in the Bt genomic database further extends the catalog of the putative VipR-regulated genes to *cry1Ae, cry1Ah, cry1Ea, cry1Fb, cry1Ia, cry1Ja, cry2Ad, cry2Ah, cry9Da*, and *cry9Ee* in strains mostly displaying a predicted toxic activity towards lepidopteran insects (Fig. 6A; Table S3).

This report, and the studies on the *cry3A* gene and on the regulator CpcR (14, 17, 18, 20), shed light on a more complex strategy of toxin production in some Bt strains, notably those used as commercial biopesticides. Furthermore, the transcription of VipR and Vip3A is stronger and more precocious in a sporulation mutant, suggesting that the entry into sporulation may be unfavorable for producing the VipR-regulated insecticidal proteins. This phenomenon may also reflect a production of toxins that is triggered by specific environmental signals. Indeed, analysis of the VipR protein sequence shows that the regulator possesses three putative regulatory domains: two phosphoenolpyruvate-sugar phosphotransferase system (PTS) regulation domains (PRD) and one EII-like binding domain (24). These domains are known to be the target of phosphorylation on histidine residues to modulate their activities or their dimerization in response to carbohydrate availability (45). VipR contains histidine residues in its PRD domains that could correspond to the phosphorylated residues in AtxA and Mga (25). Therefore, the activity of VipR might be modulated by environmental signals through the PTS so that the bacteria can produce a large set of toxins to kill their host. This hypothesis would be consistent with the widely accepted chronology of the infection (46). It is demonstrated that the Cry proteins play a major role in the first step of an infection when a susceptible insect feeds and ingests spores and crystals (46, 47). The crystals dissolve in the gut, liberating the toxins leading to toxemia and paralysis of the gut. In some susceptible insects, Cry toxins are sufficient to damage the intestinal epithelium, leading to the host’s death. However, in more resistant insects, the germination of the co-ingested spores is required to produce fatal septicemia (37). Indeed, spores and the ability to grow after germination in the insect gut have been shown to synergistically increase the insecticidal effect of the Cry proteins (48–50). This effect is due to the production of various virulence factors, mainly belonging to the PlcR regulon, that are produced by the bacterium during the stationary phase (46, 49). The role of Vip3A produced by the HD1 strain during growth has notably been evidenced in increasing significantly the toxicity of the strain towards some lepidopteran insects (4, 48). Indeed, in a S*podoptera exigua* larvae infection assay, the HD1 strain was 16× more toxic than the HD1 ∆*vip3A* strain. However, when streptomycin was added to the insect diet, the toxicity of both strains was reduced, but the HD1 strain was only 3× more toxic than the ∆*vip3A* mutant, demonstrating that the production of Vip3A during bacterial growth in the insect was critical to establish the infection (48). Using our model HD73 strain and the HD1 strain, we showed that VipR allows the production of Cry1Ac at the beginning of the stationary phase of growth, similarly to the timing of expression of Vip3A (Fig. 3A and B and 5C). This result suggests that Cry1I, the other pesticidal protein of the HD1 strain released by the bacteria during growth, may also contribute to the pathogenic effect during bacteria growth in the insect larvae, as described for Vip3A. Similarly, the early VipR-dependent production of the Cry1A and Cry2A proteins (Fig. 5C) could be involved in the virulence of the bacteria. It would be interesting to determine the precise contribution of VipR not only in producing the crystal-forming toxins but also in the complete infection cycle of Bt in its ecological niche, the insect larva.

## MATERIALS AND METHODS

### Strains and medium

The Bt *kurstaki* HD73 (51) and HD1 (52) strains belonging to serotype 3 were used in this study. The HD73 crystalliferous strain was designated as HD73, and the HD73 acrystalliferous strain as HD73 Cry^−^. *Escherichia coli* strains DH5α or NEB10 were used as the host strain for plasmid construction. *E. coli* strain ET12567 (53) was used to prepare demethylated DNA before transforming Bt by electroporation (54, 55). Bacteria were routinely grown in LB medium at 37°C for *E. coli* and at 30°C for Bt cells. Time 0 was defined as the beginning of the transition phase between the exponential and stationary phases of Bt growth. The following concentrations of antibiotics were used for Bt selection: erythromycin (5 µg/mL) and kanamycin (200 µg/mL). For *E. coli* selection, kanamycin (20 µg/mL) and ampicillin (100 µg/mL) were used. When needed, xylose (20 mM) was added to the culture.

### DNA manipulation and strain construction

The plasmids and bacterial strains used in the study are listed in Tables 1 and 2, respectively. All the DNA manipulation procedures were conducted as described in reference 24. Genomic DNA was extracted from exponentially growing *B. thuringiensis* HD1 cells using the Qiagen DNeasy blood and tissue kit. The primers (Table 3) and sequencing data were provided by Eurofins Genomics.

The pMADK (Table 1), a new version of the pMAD (30), was designed to reduce the size of the original plasmid and change the antibiotic resistance genes used to select the transformation events in *E. coli* and *Bacillus*. Briefly, the pBR222-originating ampicillin resistance gene, the pE194-originating erythromycin resistance gene, and *pre* gene were removed and replaced by a kanamycin resistance cassette that is functional both in *E. coli* and *Bacillus*, yielding the pMADK (Fig. S4). These modifications brought the advantage of increasing the number of unique restriction sites present in the MCS. Plasmid pMADK was used for gene disruption by homologous recombination (30).

### Bioinformatic analyses

The VipR boxes were identified in the plasmid and chromosomal sequences using the following sequence in its IUPAC nucleotide code (https://genome.ucsc.edu/goldenPath/help/iupac.html): 5′-YTTCHYBWATVGRKGAARWTATSTMTSCTTTW-3′ in the following sequences: the chromosome (CP004069) and pHT73 plasmid (CP004070) of the HD73 strain, in the pBMB95 (CP004875) and pBMB65 (CP004873) plasmids of the HD1 strain, and in the chromosome (CP083156), pCH_181-b (CP083158), and pCH_181-e (CP083161) plasmids of the ABTS-1857 strain. When a hit was identified, the presence of a putative −10 box located 17 nucleotides downstream of the last position of the motif was verified to confirm the identification of a VipR box.

The presence of a potential Rho-independent transcription terminator was verified using the ARNold web server (http://rssf.i2bc.paris-saclay.fr/toolbox/arnold/).

Plasmid copy number was estimated by comparing sequencing depth across the chromosome and the different plasmids in *B. thuringiensis* HD1 samples. Read alignment files (BAM) were processed using samtools coverage to compute the mean sequencing depth for each contig of the reference genome. For each sample, the relative plasmid copy number was calculated as follows: copy number = plasmid mean depth/chromosome mean depth. Contig identifiers were then mapped to annotated plasmid names using the reference GFF file, producing a consolidated plasmid copy-number matrix for all samples.

Detection of *cry* genes in *ami* genomic neighborhoods was done as follows. *ami* gene homologs were identified by blastn in *B. thuringiensis* (TaxID 1428). A 10-kb nucleotide window centered on the best-scoring hit was extracted to represent the local genomic neighborhood of each *ami* gene. These windows were screened for the presence of *cry* toxin genes using tblastn with a curated set of Cry protein sequences corresponding to the holotype of each Cry toxin (N. Crickmore, C. Berry, S. Panneerselvam, R. Mishra, T. R. Connor, and B. C. Bonning, Bacterial Pesticidal Protein Resource Center, https://www.bpprc.org.) with the following parameters: ≥95% identity and ≥95% coverage. Only full-length matches were retained to exclude truncated or partial *cry* variants. To determine whether *cry* genes are present more frequently around *ami* genes than expected by chance, the same detection procedure was applied to a genome-wide background. Complete genomes were partitioned into 10-kb non-overlapping windows covering all chromosomal and plasmid sequences, and the frequency of *cry*-positive windows in this genome-wide data set was used as a reference rate for comparison with *ami*-centered windows.

Detection of the VipR boxes upstream of *cry* genes was done as follows. A comprehensive TBLASTN search was conducted using a curated database of all Cry protein holotypes to identify *cry* genes in the genomes of *Bacillus thuringiensis* (TaxID 1428). Parameters used for TBLASTN were the following: *E* value < 1e−5, pident ≥ 95%, and qcovs ≥ 95%. Using samtools faidx with precise genomic coordinates provided by tblastn results, a promoter region of 2,100 bp upstream of the translation initiation site was extracted, generating the *cry* promoter data set. To screen the data set for sequences matching the VipR box consensus motif derived from WebLogo analysis, a pattern-matching approach based on regular expressions was employed. The degenerate consensus sequence was defined according to the IUPAC nomenclature as follows: 5′-YTTCHYBWATVGRKGAARWTATSTMTSCTTTW-3′. The computational screening was performed using a custom Python 3 script. The script dynamically converted the IUPAC degenerate sequence into an equivalent regular expression, mapping each ambiguity code to its respective alternative bases (e.g., Y mapped to [CT] and R mapped to [AG]). The *cry* promoter data set in FASTA format was then iteratively parsed. Exact matches within each sequence were identified on the forward strand using the Python re module (the re.finditer function). The localized motifs were compiled into a tab-delimited output file recording the sequence identifier, the exact 0-indexed start position, and the retrieved nucleotide sequence. Results were compiled and summarized by Cry type (e.g., Cry1Aa1 and Cry1Ab1) and by their organism of origin (strain name and serotype when defined).

### β-Galactosidase assay

The β-galactosidase activity was monitored using a qualitative method. Cells were streaked on LB plates containing X-gal (50 μg/mL) and xylose 20 mM and incubated at 30°C for 24 h, followed by an incubation at 20°C for another 24 h. The blue coloration reflects the activity of the β-galactosidase.

### mRNA extraction and RT-PCR

RNA was extracted from the Bt HD73 strains grown at 30°C in LB medium and harvested at T2. RNA samples were prepared as described (20) and stored at −80°C before use. The cDNA was generated using random hexamer primers and the Maxima H Minus Reverse Transcriptase (Thermo Scientific) on 1 μg of total RNA according to the manufacturer’s instructions (+RT). For each RNA sample, a negative control reaction was performed in the absence of the reverse transcriptase (−RT). A subsequent PCR was realized with the RTamid-cry1A-F/RTamid-cry1A-R primer pair on each sample (+RT and −RT) using a standard Taq polymerase (NEB). A PCR positive control was realized on HD73 genomic DNA. The production of an amplicon was visualized on 0.8% agarose gel in TAE 0.5× buffer.

### SDS-PAGE and Western blot assays

For SDS-PAGE analysis, Bt HD73 strains were grown in LB medium at 30°C in agitated cultures. For each time point, 5 mL culture was collected. Bacteria were separated from the growth medium by centrifugation at 5,000 × *g* for 10 min. The bacterial cell pellet was resuspended in 500 µL H_2_O and the suspension was treated with lysozyme (0.1 mg/mL) at 30°C for 30 min. Cells were then disrupted with glass beads in a FastPrep 24 (MP Biomedical). The total cell extract was obtained after the sedimentation of the beads. An equal volume of sample (3 µL for SDS-PAGE and 0.3 µL for Western blot) was separated using SDS-PAGE on a 10% polyacrylamide gel. Gels were either stained with the Readyblue protein gel stain (Sigma) or subjected to Western blot assays. Total protein extracts of the HD1 strains were produced as follows: strains were grown on LB plates for 2 days at 30°C, followed by 6 days at 20°C. Colonies were scraped from plates, resuspended in 500 µL H_2_O, and treated as described above.

Western blot assays were performed as described in reference 24 using a primary antiserum raised against crystal proteins of the Bt *aizawai* 7.29 strain (56) available in the lab and used at the 1:20,000 dilution and a mouse anti-Vip3Aa monoclonal antibody used at the 1:10,000 dilution.

### Microscopy

Cells were observed with a Zeiss AxioObserver.Z1 inverted fluorescence microscope equipped with a Zeiss AxioCam MRm digital camera. Images were processed with the Zeiss ZEN 2.0 software package.

### LC-MS/MS analysis

Five micrograms of a total protein extract of the HD1 ∆*spo0A* strain was loaded onto a 10% polyacrylamide gel, and the gel was run for a short migration. Proteomics analyses were performed on the PAPPSO platform (http://pappso.inra.fr). The proteins were in-gel-digested with trypsin as described in reference 57. All peptide samples were analyzed using a timsTOF Pro mass spectrometer coupled with a nanoElute liquid chromatography system (Bruker Daltonik GmbH, Germany). Peptides were directly injected and separated onto an Aurora C18 column (75 μm i.d. × 250 mm, 1.6 μm, 120 Å, Ion Opticks, Australia) in buffer A (0.1% formic acid and 2% acetonitrile). The peptides were eluted with the following gradient: 2–5% of buffer B (0.1% formic acid in 100% acetonitrile) for 1 min, 5–13% of buffer B for 18 min, 13–19% of buffer B for 7 min, and 19–22% of buffer B for 4 min. The column temperature was set to 50°C. Peptides were introduced into the mass spectrometer via a CaptiveSpray nanoelectrospray ion source (Bruker Daltonik GmbH) at an electrospray voltage of 1.6 kV, an ion source temperature of 180°C, and a dry gas of 3 L/min. Data collection was carried out using DDA-PASEF mode. The mobility scan range used was 0.7–1.10 V·s cm^−2^ (1/K0), the collisional energy increased linearly from 20.0 eV at 0.6 V·s cm^−2^ (1/K0) to 59.0 eV at 1.60 V·s cm^−2^ (1/K0). Both MS and MS/MS spectra were recorded within a scan range of 100–1,700 *m/z*. TIMS accumulation time was set to 180 ms, with a target precursor intensity of 14,000 arbitrary units (au) and a minimum threshold of 1,000 au. Absolute intensity thresholds were set to 10 for mass spectra peak detection and 5,000 for mobilogram peak detection. Protein identification was performed by querying MS/MS data against a FASTA format database of the Bt *kurstaki* HD1 (GCF_000717535.1) together with a custom contaminant database using the i2MassChroQ software (version 1.0.19, http://pappso.inrae.fr/). The identified proteins were filtered with a minimum of two different peptides, with a peptide *E* value <10^−2^ and protein *E* value <10^−4^.

## SUPPLEMENTAL MATERIAL

Supplemental figures and tables mbio.01592-26-s0001.pdf Fig. S1 to S6 and Tables S1 to S4.

ASM does not own the copyrights to Supplemental Material that may be linked to, or accessed through, an article. The authors have granted ASM a non-exclusive, world-wide license to publish the Supplemental Material files. Please contact the corresponding author directly for reuse.
