# Supplementary material for: Paradigm shift for cry gene expression in Bacillus thuringiensis
Source: mBio. 2026 Aug 5;17(9):e01592-26. doi: 10.1128/mbio.01592-26 (PMC13556251; doi:10.1128/mbio.01592-26)
Supplement: Supplementary file 1 [file mbio.01592-26-s0001.pdf]

Paradigm shift for *cry* gene expression in *Bacillus thuringiensis*

Emilie Verplaetse[a,#], Alicia Nevers [a], Leyla Slamti[a,#], Didier Lereclus[a]

[a] Université Paris-Saclay, INRAE, AgroParisTech, Micalis Institute, 78350, Jouy-en-Josas, France

Running title: Sporulation-independent expression of *cry* genes

#Corresponding authors: emilie.verplaetse@inrae.fr , leyla.slamti@inrae.fr

Key-words: biopesticide, plasmid, gene regulation, transcription, insecticidal, noctuidae

Figure S1

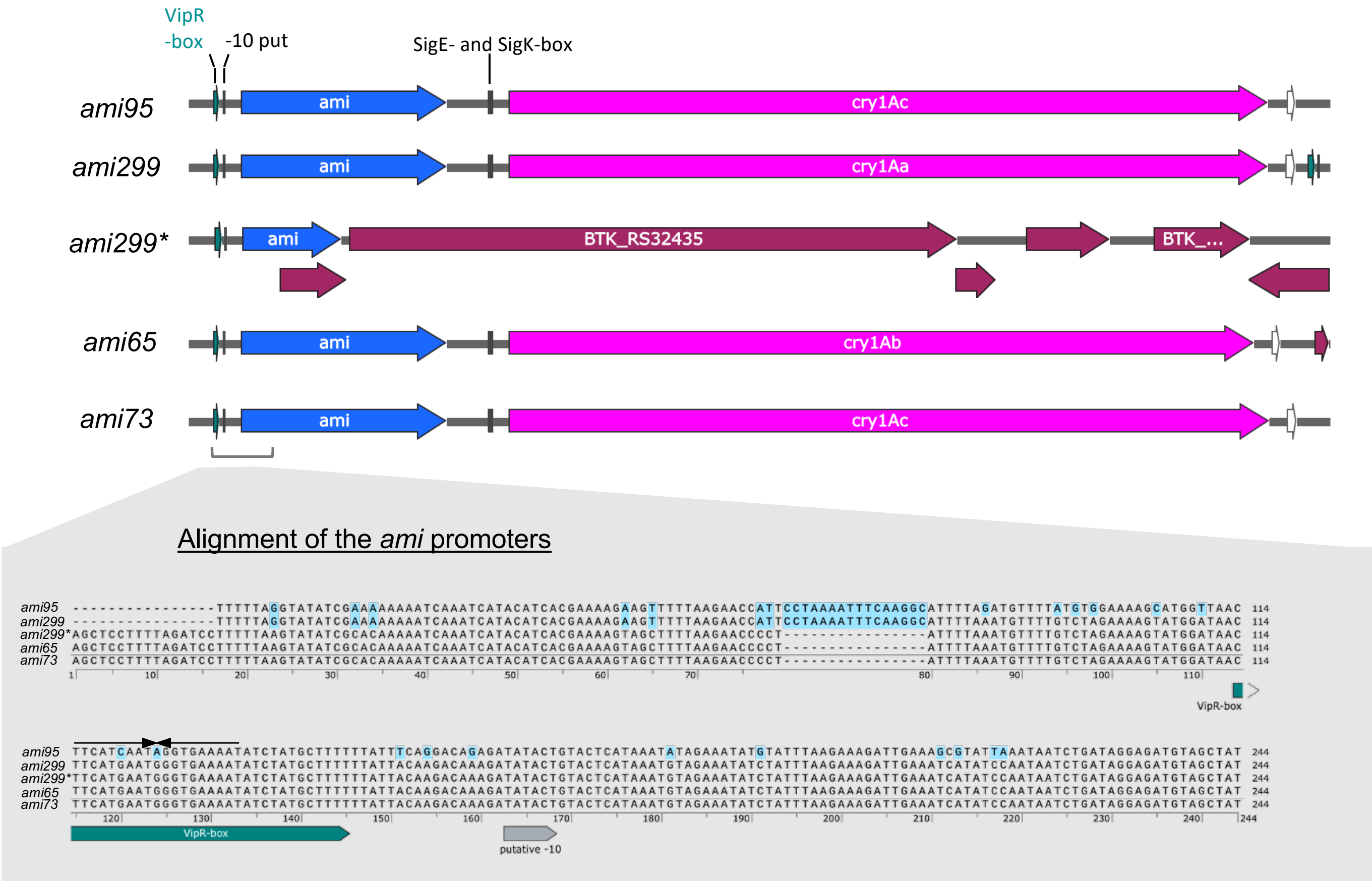

Fig. S1- Schematic of the *ami-cry1A* loci of the Bt HD1 and HD73 strains. The SigE and SigK boxes, and the VipR-regulated promoters are indicated. Putative terminators are shown in white. An alignment of the DNA sequences of the *ami* promoters containing the VipR box is shown at the bottom. Black arrows indicate the position of the imperfect palindrome.

Figure S2

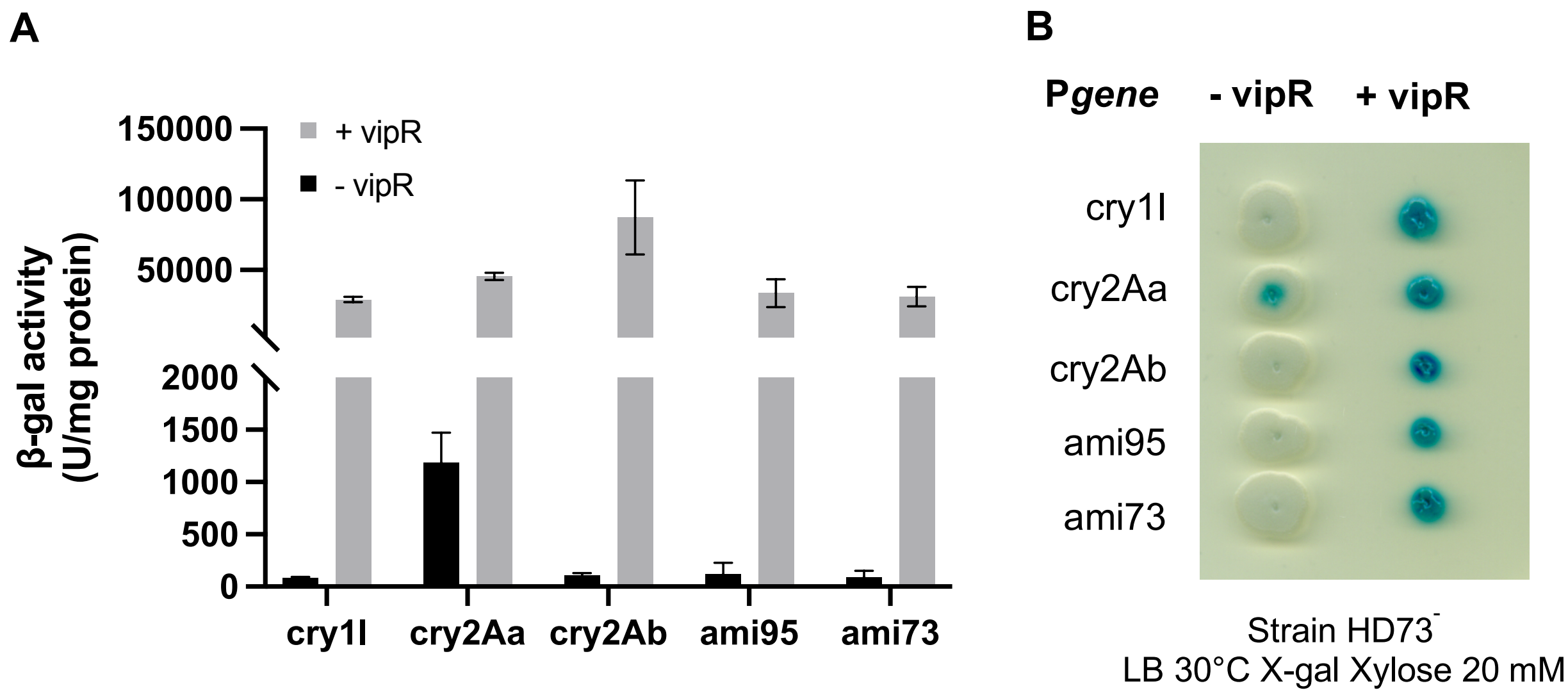

Fig. S2- VipR controls the expression of the insecticidal protein genes containing a VipR box in their promoter. **A**. ß-galactosidase activity of the *B. thuringiensis* HD73 Cry- strains harboring the plasmids pP_{gene}-*lacZ* and the pHT-P_{xyl}-*vipR* or the pHT-P_{xyl}. The name of the gene whose promoter is fused to *lacZ* is indicated at the bottom of the columns. Strains were patched on LB medium containing xylose 20 mM and the ß-galactosidase activity was assayed after 24 h of growth at 30°C. The activity was measured as previously described and expressed as units per milligram of protein (Perchat S, Dubois T, Zouhir S, Gominet M, Poncet S, Lemy C, Aumont-Nicaise M, Deutscher J, Gohar M, Nessler S, Lereclus D. 2011. A cell-cell communication system regulates protease production during sporulation in bacteria of the *Bacillus cereus* group. Mol Microbiol 82:619-33). Data are the mean ± SD, n=3. B. The strains HD73 Cry- harboring the plasmids pP_{gene}-*lacZ* and the pHT-P_{xyl}-*vipR* (column « + vipR ») or the pHT-P_{xyl} (column « - vipR ») described in A. were patched on LB medium containing X-gal (50 µg/mL) and xylose 20 mM and grown at 30°C for 24 h. The picture was taken after an additional 24 h of growth at room temperature and is representative of at least 3 experiments.

Figure S3

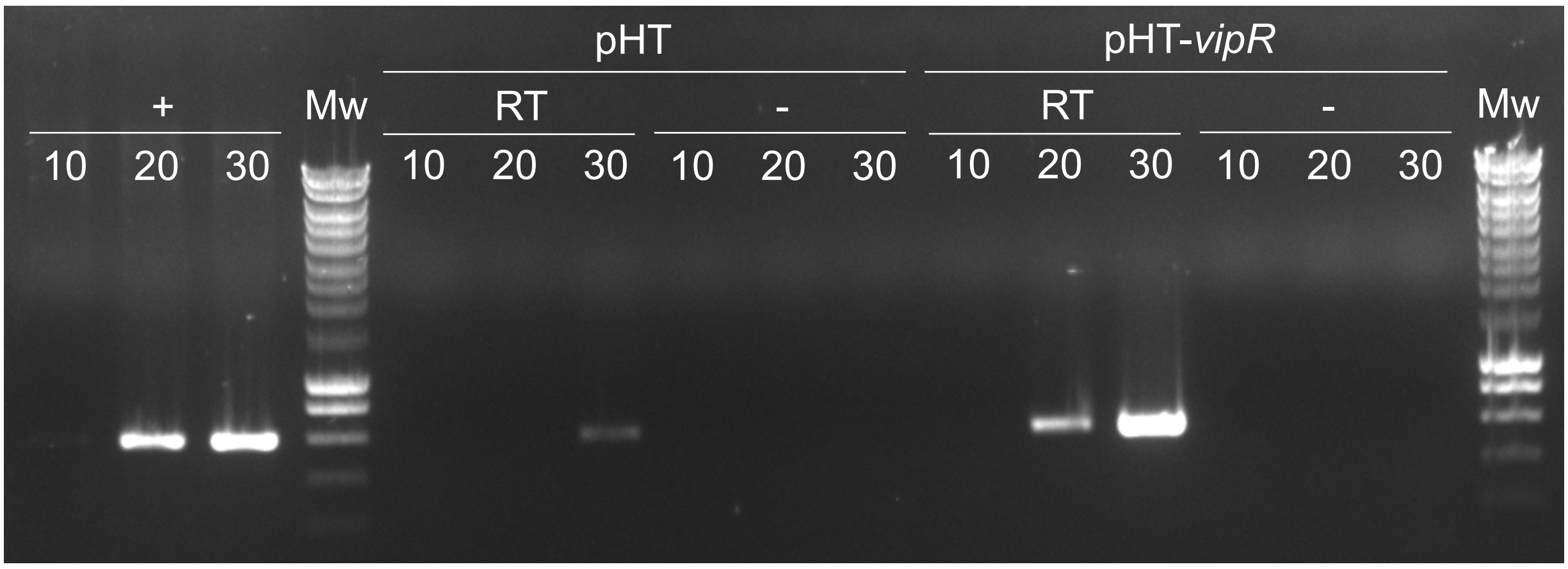

Fig. S3- Analysis of the *ami73-cry1Ac* operon in the HD73. Agarose gel electrophoresis analysis of the production of a 600-bp PCR product obtained with the RTamid-cry1A-F and RTamid-cry1A-R primer pair and HD73 genomic DNA as the DNA template (lanes +), or HD73 (pHT) and HD73 (pHT-vipR) RNA treated (lanes RT), or not (lanes -), with the reverse transcriptase. 10, 20 or 30 PCR cycles were performed for each sample as indicated above the wells. RNA was collected 2 hours after the onset of stationary phase from bacteria grown in LB medium at 30°C. Mw, SmartLadder® DNA molecular weight ladder. The experiments were repeated twice.

Figure S4

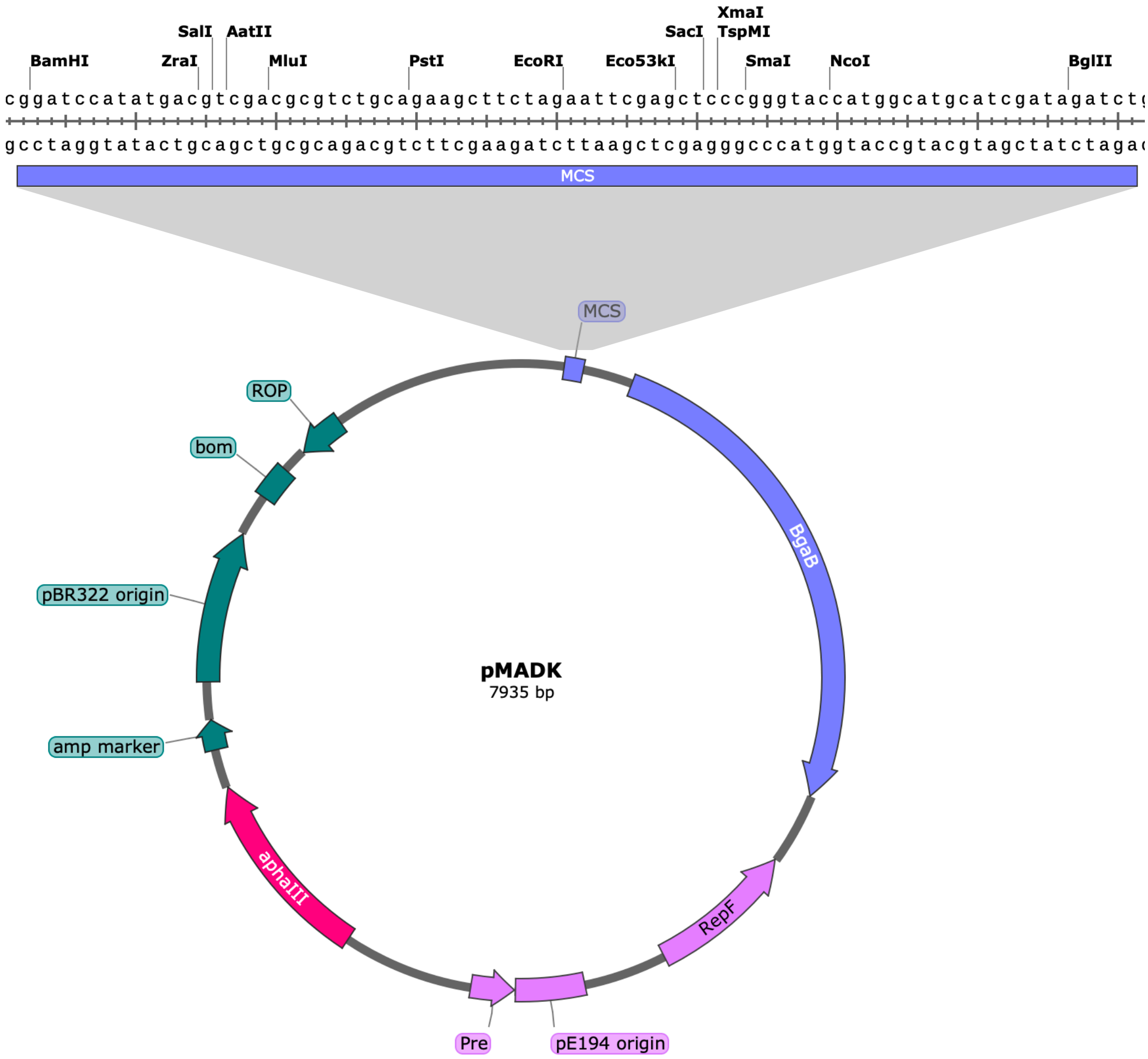

Fig. S4- Schematic representing the pMADK and focus on the DNA sequence containing the multiple cloning site (MCS). Unique restriction sites are indicated.

Figure S5

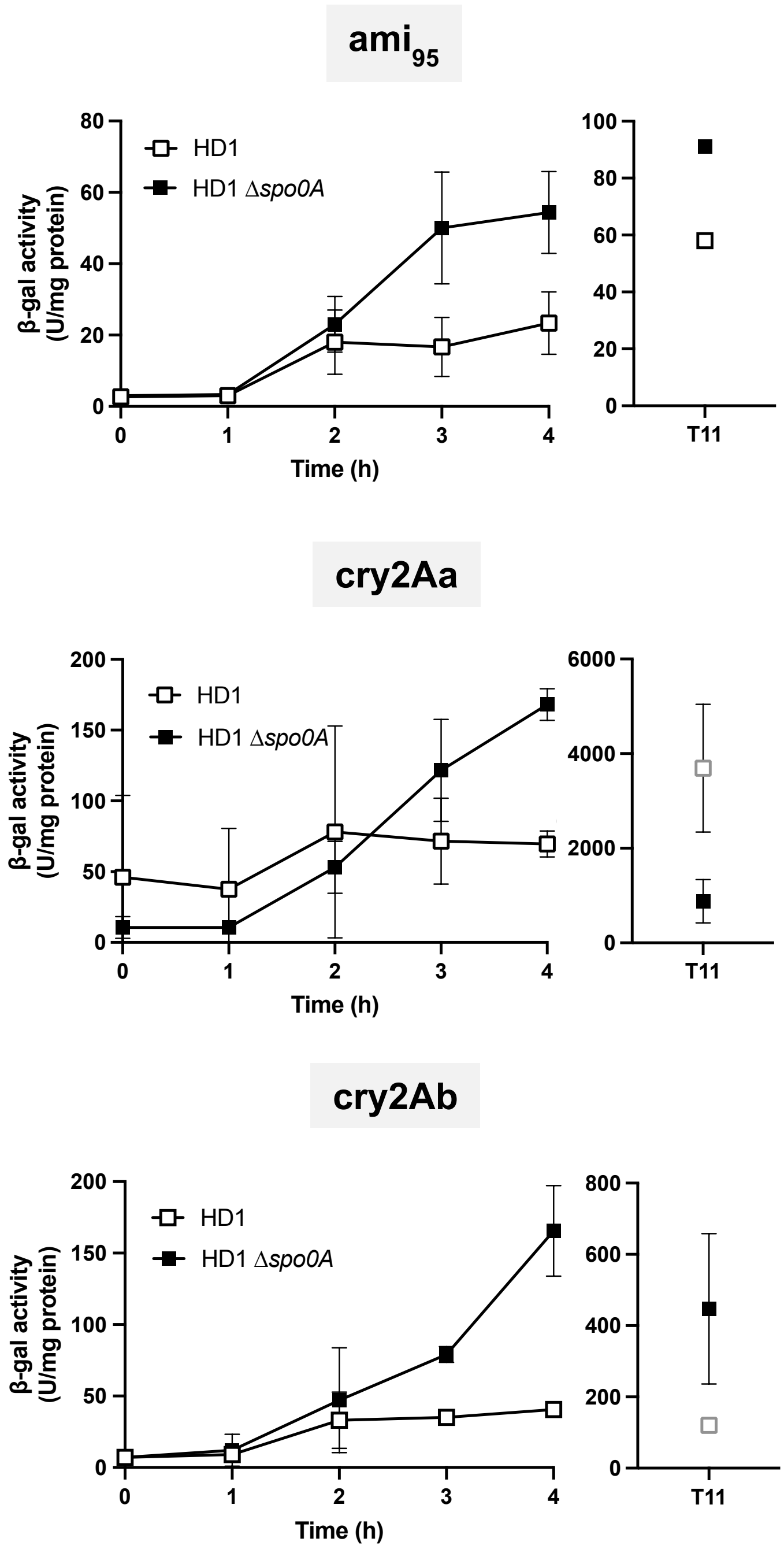

Fig. S5- β-Galactosidase activity of the HD1 and HD1 Δ*spo0A* strains carrying the pHT-P_{ami95}, pHT-P_{cry2Aa} or the pHT-P_{cry2Ab} plasmid. Bacteria were grown in LB at 30°C. Time 0 corresponds to the entry of the bacteria into stationary phase. The activity was measured as described in Fig. S2 and expressed as units per milligram of protein. Data are the mean ± SD, n=3 for *ami95*, n=2 for *cry2Aa* and *cry2Ab.* The activity was measured as described in Fig. S2 and expressed as units per milligram of protein.

Figure S6

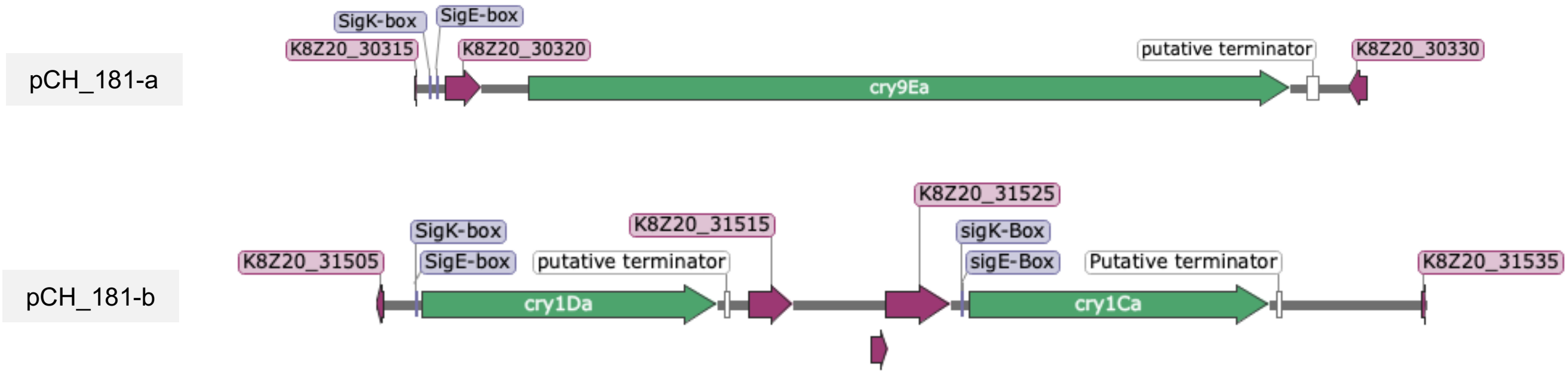

Fig. S6- Schematic of the *cry9Ea, cry1Ca and cry1Da* loci of the Bt *aizawai* ABTS-1857 strain. The SigE- and SigK-boxes are indicated. Putative terminators are shown in white.

Table S1: List of the insecticidal protein genes of the Bt *kurstaki* HD1, *kurstaki* HD73 and *aizawai* ABTS-1857 strains and their plasmid location.

Strain	Plasmid name	Insecticidal protein transcription units
kurstaki HD1	pBMB299	*vip3Aa, cry1Ia, ami299-cry1Aa, orf1-orf2-cry2Aa, cry2Ab*
	pBMB95	*ami95-cry1Ac*
	pBMB65	*ami65-cry1Ab*
kurstaki HD73	pHT73	*ami73-cry1Ac*
aizawai ABTS-1857	pCH_181-a	*cry9Ea*
	pCH_181-b	*vip3Aa, cry1Ia,* ami_{pCH_181-b} *-cry1Aa, cry2Ab, cry1Ca, cry1Da*
	pCH_181-e	ami_{pCH_181-e} *-cry1Ab*

Table S2: Estimation of the copy number of the HD1 plasmids

	HD1				WT
sample	contig	plasmid_name	length_bp	mean_depth	ratio_vs_chr
HD1_WT	NZ_CP004870.1	Chromosome	5631672	191,02	1
HD1_WT	NZ_CP004883.1	pBMB14	14721	697,14	3,6496
HD1_WT	NZ_CP004882.1	pBMBLin15	14870	704,25	3,6869
HD1_WT	NZ_CP004871.1	pBMB46	46634	1003,09	5,2513
HD1_WT	NZ_CP004872.1	pBMB64	64522	296,76	1,5536
HD1_WT	NZ_CP004873.1	pBMB65	65873	255,76	1,3389
HD1_WT	NZ_CP004874.1	pBMB74	74480	339,51	1,7774
HD1_WT	NZ_CP004875.1	pBMB95	95983	376,23	1,9696
HD1_WT	NZ_CP004876.1	pBMB299	299843	326,55	1,7095
HD1_WT	NZ_CP004877.1	pBMB431	431546	144,99	0,7591
HD1_WT	NZ_CP004878.1	pBMB2062	2062	734,18	3,8436
HD1_WT	NZ_CP004879.1	pBMB7635	7635	650,7	3,4065
HD1_WT	NZ_CP004880.1	pBMB8240	8240	396,18	2,074
HD1_WT	NZ_CP004881.1	pBMB8513	8513	1303,14	6,8222

Column	Meaning
sample	Name of the sample
contig	Identifier of the DNA segment in the reference genome
plasmid_name	Name of the corresponding plasmid
length_bp	Contig size in base pairs
mean_depth	Average coverage (average number of reads per base)
ratio_vs_chr	Ratio between plasmid coverage and chromosome coverage

Table S3: *cry* gene promoters containing a VipR box.

Gene holotype	Genbank accession number	window_start	window_end	Motif position	Motif sequence	pident	Bacillus thuringiensis serovar	Strain name	Plasmid name
**Bacillus thuringiensis serovar kurstaki**									
Cry1Aa1	CP083118.1	42040	44139	723	CTTCATCAATAGGTGAAAATATCTATGCTTTT	100.0	Bacillus thuringiensis serovar kurstaki	SA11	pCH_164-b
Cry1Ia1	CP083118.1	38003	40102	1781	TTTCACCTATCGATGAAAATATCTCTGCTTTT	100.0	Bacillus thuringiensis serovar kurstaki	SA11	pCH_164-b
Cry2Aa1	CP083118.1	26622	28721	25	CTTCCTCTATAGAGGAAGATATCTATGCTTTT	100.0	Bacillus thuringiensis serovar kurstaki	SA11	pCH_164-b
Cry2Ab1	CP083118.1	51786	53885	1300	CTTCTTCTATAGGTGAAGATATCTATGCTTTT	100.0	Bacillus thuringiensis serovar kurstaki	SA11	pCH_164-b
Cry1Aa1	CP083103.1	114499	116598	723	CTTCATCAATAGGTGAAAATATCTATGCTTTT	100.0	Bacillus thuringiensis serovar kurstaki	ABTS-351	pCH_183-b
Cry1Ac1	CP083103.1	65690	67789	723	CTTCATGAATGGGTGAAAATATCTATGCTTTT	100.0	Bacillus thuringiensis serovar kurstaki	ABTS-351	pCH_183-b
Cry1Ia1	CP083103.1	110462	112561	1781	TTTCACCTATCGATGAAAATATCTCTGCTTTT	100.0	Bacillus thuringiensis serovar kurstaki	ABTS-351	pCH_183-b
Cry2Aa1	CP083103.1	99081	101180	25	CTTCCTCTATAGAGGAAGATATCTATGCTTTT	100.0	Bacillus thuringiensis serovar kurstaki	ABTS-351	pCH_183-b
Cry2Ab1	CP083103.1	124245	126344	1300	CTTCTTCTATAGGTGAAGATATCTATGCTTTT	100.0	Bacillus thuringiensis serovar kurstaki	ABTS-351	pCH_183-b
Cry1Aa1	CP158700.1	122680	124779	723	CTTCATCAATAGGTGAAAATATCTATGCTTTT	100.0	Bacillus thuringiensis serovar kurstaki	HD-1	unnamed2
Cry1Ac1	CP158700.1	73871	75970	723	CTTCATGAATGGGTGAAAATATCTATGCTTTT	100.0	Bacillus thuringiensis serovar kurstaki	HD-1	unnamed2
Cry1Ia1	CP158700.1	118643	120742	1781	TTTCACCTATCGATGAAAATATCTCTGCTTTT	100.0	Bacillus thuringiensis serovar kurstaki	HD-1	unnamed2
Cry2Aa1	CP158700.1	107262	109361	25	CTTCCTCTATAGAGGAAGATATCTATGCTTTT	100.0	Bacillus thuringiensis serovar kurstaki	HD-1	unnamed2
Cry2Ab1	CP158700.1	132426	134525	1300	CTTCTTCTATAGGTGAAGATATCTATGCTTTT	100.0	Bacillus thuringiensis serovar kurstaki	HD-1	unnamed2
Cry1Aa1	CP072692.1	106044	108143	723	CTTCATCAATAGGTGAAAATATCTATGCTTTT	100.0	Bacillus thuringiensis serovar kurstaki	IMBL-B9	pKBT365
Cry1Ah1	CP072692.1	206223	208322	784	CTTCATCAATAGGTGAAAATATCTATGCTTTT	100.0	Bacillus thuringiensis serovar kurstaki	IMBL-B9	pKBT365
Cry1Ia1	CP072692.1	102007	104106	1781	TTTCACCTATCGATGAAAATATCTCTGCTTTT	100.0	Bacillus thuringiensis serovar kurstaki	IMBL-B9	pKBT365
Cry2Aa1	CP072692.1	91136	93235	25	CTTCCTCTATAGAGGAAGATATCTATGCTTTT	100.0	Bacillus thuringiensis serovar kurstaki	IMBL-B9	pKBT365
Cry2Ab1	CP072692.1	115790	117889	1300	CTTCTTCTATAGGTGAAGATATCTATGCTTTT	100.0	Bacillus thuringiensis serovar kurstaki	IMBL-B9	pKBT365
Cry2Ah1	CP072692.1	141139	143238	811	CTTCTTCTATAGGTGAAGATATCTATGCTTTT	100.0	Bacillus thuringiensis serovar kurstaki	IMBL-B9	pKBT365
Cry1Aa1	CP116317.1	142300	144399	723	CTTCATCAATAGGTGAAAATATCTATGCTTTT	100.0	Bacillus thuringiensis serovar kurstaki	Lip	pLip300
Cry1Ac1	CP116317.1	176572	178671	344	CTTCATGAATGGGTGAAAATATCTATGCTTTT	100.0	Bacillus thuringiensis serovar kurstaki	Lip	pLip300
Cry1Ia1	CP116317.1	146337	148436	1781	TTTCACCTATCGATGAAAATATCTCTGCTTTT	100.0	Bacillus thuringiensis serovar kurstaki	Lip	pLip300
Cry2Aa1	CP116317.1	157718	159817	25	CTTCCTCTATAGAGGAAGATATCTATGCTTTT	100.0	Bacillus thuringiensis serovar kurstaki	Lip	pLip300
Cry2Ab1	CP116317.1	132554	134653	1300	CTTCTTCTATAGGTGAAGATATCTATGCTTTT	100.0	Bacillus thuringiensis serovar kurstaki	Lip	pLip300
Cry1Aa1	CP007615.1	205362	207461	723	CTTCATCAATAGGTGAAAATATCTATGCTTTT	100.0	Bacillus thuringiensis serovar kurstaki	YBT-1520	pBMB293
Cry1Ia1	CP007615.1	209399	211498	1781	TTTCACCTATCGATGAAAATATCTCTGCTTTT	100.0	Bacillus thuringiensis serovar kurstaki	YBT-1520	pBMB293
Cry2Aa1	CP007615.1	220780	222879	25	CTTCCTCTATAGAGGAAGATATCTATGCTTTT	100.0	Bacillus thuringiensis serovar kurstaki	YBT-1520	pBMB293
Cry2Ab1	CP007615.1	195616	197715	1300	CTTCTTCTATAGGTGAAGATATCTATGCTTTT	100.0	Bacillus thuringiensis serovar kurstaki	YBT-1520	pBMB293
Cry1Aa1	CP004861.1	232227	234326	723	CTTCATCAATAGGTGAAAATATCTATGCTTTT	100.0	Bacillus thuringiensis serovar kurstaki	YBT-1520	pBMB293
Cry1Ia1	CP004861.1	236264	238363	1781	TTTCACCTATCGATGAAAATATCTCTGCTTTT	100.0	Bacillus thuringiensis serovar kurstaki	YBT-1520	pBMB293
Cry2Aa1	CP004861.1	247645	249744	25	CTTCCTCTATAGAGGAAGATATCTATGCTTTT	100.0	Bacillus thuringiensis serovar kurstaki	YBT-1520	pBMB293
Cry2Ab1	CP004861.1	222481	224580	1300	CTTCTTCTATAGGTGAAGATATCTATGCTTTT	100.0	Bacillus thuringiensis serovar kurstaki	YBT-1520	pBMB293
Cry1Aa1	CP004876.1	233798	235897	723	CTTCATGAATGGGTGAAAATATCTATGCTTTT	100.0	Bacillus thuringiensis serovar kurstaki	HD1	pBMB299
Cry1Ia1	CP004876.1	237835	239934	1781	TTTCACCTATCGATGAAAATATCTCTGCTTTT	100.0	Bacillus thuringiensis serovar kurstaki	HD1	pBMB299
Cry2Aa1	CP004876.1	249299	251398	25	CTTCCTCTATAGAGGAAGATATCTATGCTTTT	100.0	Bacillus thuringiensis serovar kurstaki	HD1	pBMB299
Cry2Ab1	CP004876.1	224044	226143	1300	CTTCTTCTATAGGTGAAGATATCTATGCTTTT	100.0	Bacillus thuringiensis serovar kurstaki	HD1	pBMB299
Cry1Ab1	CP158704.1	13272	15371	723	CTTCATGAATGGGTGAAAATATCTATGCTTTT	100.0	Bacillus thuringiensis serovar kurstaki	HD-1	unnamed6
Cry1Ab1	CP116324.1	75253	77352	723	CTTCATGAATGGGTGAAAATATCTATGCTTTT	100.0	Bacillus thuringiensis serovar kurstaki	Lip	pLip97
Cry1Ac1	CP116324.1	16408	18507	723	CTTCATGAATGGGTGAAAATATCTATGCTTTT	100.0	Bacillus thuringiensis serovar kurstaki	Lip	pLip97
Cry1Ab1	CP083107.1	19027	21126	723	CTTCATGAATGGGTGAAAATATCTATGCTTTT	100.0	Bacillus thuringiensis serovar kurstaki	ABTS-351	pCH_183-f
Cry1Ab1	CP083120.1	19738	21837	723	CTTCATGAATGGGTGAAAATATCTATGCTTTT	100.0	Bacillus thuringiensis serovar kurstaki	SA11	pCH_164-d
Cry1Ab1	CP004873.1	52073	54172	723	CTTCATGAATGGGTGAAAATATCTATGCTTTT	100.0	Bacillus thuringiensis serovar kurstaki	HD1	pBMB65
Cry1Ac1	CP004070.1	7049	9148	723	CTTCATGAATGGGTGAAAATATCTATGCTTTT	100.0	Bacillus thuringiensis serovar kurstaki	HD73	pHT73
Cry1Ac1	CP007613.1	43543	45642	723	CTTCATGAATGGGTGAAAATATCTATGCTTTT	100.0	Bacillus thuringiensis serovar kurstaki	YBT-1520	pBMB69
Cry1Ac1	CP004875.1	90898	92997	723	CTTCATCAATAGGTGAAAATATCTATGCTTTT	100.0	Bacillus thuringiensis serovar kurstaki	HD1	pBMB95
Cry1Ea1	CP072694.1	44102	46201	704	CTTCATGAATGGGTGAAAATATCTATGCTTTT	100.0	Bacillus thuringiensis serovar kurstaki	IMBL-B9	pKBT91
**Bacillus thuringiensis serovar aizawai**									
Cry1Aa1	CP116329.1	289977	292076	723	CTTCATCAATAGGTGAAAATATCTATGCTTTT	100.0	Bacillus thuringiensis serovar aizawai	BLB1	pBLB1_317
Cry1Ac1	CP116329.1	21462	23561	723	CTTCATGAATGGGTGAAAATATCTATGCTTTT	100.0	Bacillus thuringiensis serovar aizawai	BLB1	pBLB1_317
Cry1Ia1	CP116329.1	294014	296113	1781	TTTCACCTATCGATGAAAATATCTCTGCTTTT	100.0	Bacillus thuringiensis serovar aizawai	BLB1	pBLB1_317
Cry2Aa1	CP116329.1	305395	307494	25	CTTCCTCTATAGAGGAAGATATCTATGCTTTT	100.0	Bacillus thuringiensis serovar aizawai	BLB1	pBLB1_317
Cry2Ab1	CP116329.1	280231	282330	1300	CTTCTTCTATAGGTGAAGATATCTATGCTTTT	100.0	Bacillus thuringiensis serovar aizawai	BLB1	pBLB1_317
Cry1Aa1	CP059978.1	42710	44809	723	CTTCATCAATAGGTGAAAATATCTATGCTTTT	100.0	Bacillus thuringiensis serovar aizawai	G03	pWK295
Cry1Ia1	CP059978.1	48413	50512	1781	TTTCACCTATCGATGAAAATATCTCTGCTTTT	100.0	Bacillus thuringiensis serovar aizawai	G03	pWK295
Cry2Ab1	CP059978.1	32964	35063	1300	CTTCTTCTATAGGTGAAGATATCTATGCTTTT	100.0	Bacillus thuringiensis serovar aizawai	G03	pWK295
Cry1Aa1	CP083146.1	102569	104668	723	CTTCATCAATAGGTGAAAATATCTATGCTTTT	100.0	Bacillus thuringiensis serovar aizawai	GC-91	pCH_186-b
Cry1Ia1	CP083146.1	96866	98965	1781	TTTCACCTATCGATGAAAATATCTCTGCTTTT	100.0	Bacillus thuringiensis serovar aizawai	GC-91	pCH_186-b
Cry2Ab1	CP083146.1	112315	114414	1300	CTTCTTCTATAGGTGAAGATATCTATGCTTTT	100.0	Bacillus thuringiensis serovar aizawai	GC-91	pCH_186-b
Cry1Aa1	CP083158.1	106131	108230	723	CTTCATCAATAGGTGAAAATATCTATGCTTTT	100.0	Bacillus thuringiensis serovar aizawai	ABTS-1857	pCH_181-b
Cry1Ia1	CP083158.1	102094	104193	1781	TTTCACCTATCGATGAAAATATCTCTGCTTTT	100.0	Bacillus thuringiensis serovar aizawai	ABTS-1857	pCH_181-b
Cry2Ab1	CP083158.1	115877	117976	1300	CTTCTTCTATAGGTGAAGATATCTATGCTTTT	100.0	Bacillus thuringiensis serovar aizawai	ABTS-1857	pCH_181-b
Cry1Aa1	CP083087.1	249727	251826	723	CTTCATCAATAGGTGAAAATATCTATGCTTTT	100.0	Bacillus thuringiensis serovar aizawai	B401	pP05_2-b
Cry1Ia1	CP083087.1	244024	246123	1781	TTTCACCTATCGATGAAAATATCTCTGCTTTT	100.0	Bacillus thuringiensis serovar aizawai	B401	pP05_2-b
Cry2Ab1	CP083087.1	259473	261572	1300	CTTCTTCTATAGGTGAAGATATCTATGCTTTT	100.0	Bacillus thuringiensis serovar aizawai	B401	pP05_2-b
Cry1Ab1	CP083161.1	17736	19835	724	CTTCATGAATGGGTGAAAATATCTATGCTTTT	100.0	Bacillus thuringiensis serovar aizawai	ABTS-1857	pCH_181-e
Cry1Ab1	CP083091.1	19620	21719	723	CTTCATGAATGGGTGAAAATATCTATGCTTTT	100.0	Bacillus thuringiensis serovar aizawai	B401	pP05_2-f
Cry1Ac1	CP083147.1	25043	27142	723	CTTCATGAATGGGTGAAAATATCTATGCTTTT	100.0	Bacillus thuringiensis serovar aizawai	GC-91	pCH_186-c

Gene holotype	Genbank accession number	window_start	window_end	Motif position	Motif sequence	pident	Bacillus thuringiensis serovar	Strain name	Plasmid name
**Bacillus thuringiensis serovar thuringiensis**									
Cry1Aa1	CP043233.1	202792	204891	723	CTTCATCAATAGGTGAAAATATCTATGCTTTT	100.0	Bacillus thuringiensis serovar thuringiensis	Bt-GS57	unnamed2
Cry1Ia1	CP043233.1	206829	208928	1781	TTTCACCTATCGATGAAAATATCTCTGCTTTT	100.0	Bacillus thuringiensis serovar thuringiensis	Bt-GS57	unnamed2
Cry2Ab1	CP043233.1	193046	195145	1300	CTTCTTCTATAGGTGAAGATATCTATGCTTTT	100.0	Bacillus thuringiensis serovar thuringiensis	Bt-GS57	unnamed2
Cry1Aa1	CP004136.1	269098	271197	724	CTTCATCAATAGGTGAAAATATCTATGCTTTT	100.0	Bacillus thuringiensis serovar thuringiensis	IS5056	pIS56-285
Cry1Ia1	CP004136.1	273135	275234	1781	TTTCACCTATCGATGAAAATATCTCTGCTTTT	100.0	Bacillus thuringiensis serovar thuringiensis	IS5056	pIS56-285
Cry2Ab1	CP004136.1	259353	261452	1300	CTTCTTCTATAGGTGAAGATATCTATGCTTTT	100.0	Bacillus thuringiensis serovar thuringiensis	IS5056	pIS56-285
Cry1Ab1	CP004131.1	8894	10993	723	CTTCATGAATGGGTGAAAATATCTATGCTTTT	100.0	Bacillus thuringiensis serovar thuringiensis	IS5056	pIS56-63
Cry1Ab1	CP020758.1	8994	11093	725	CTTCATGAATGGGTGAAAATATCTATGCTTTT	100.0	Bacillus thuringiensis serovar thuringiensis	ATCC 10792	pLDW-19
Cry1Ab1	CP021065.1	32177	34276	723	CTTCATGAATGGGTGAAAATATCTATGCTTTT	100.0	Bacillus thuringiensis serovar thuringiensis	ATCC 10792	poh4
**Bacillus thuringiensis serovar galleriae**									
Cry1Aa1	CP010091.1	246419	248518	723	CTTCATCAATAGGTGAAAATATCTATGCTTTT	100.0	Bacillus thuringiensis serovar galleriae	HD-29	pBMB267
Cry1Ia1	CP010091.1	250456	252555	1781	TTTCACCTATCGATGAAAATATCTCTGCTTTT	100.0	Bacillus thuringiensis serovar galleriae	HD-29	pBMB267
Cry2Ab1	CP010091.1	236673	238772	1300	CTTCTTCTATAGGTGAAGATATCTATGCTTTT	100.0	Bacillus thuringiensis serovar galleriae	HD-29	pBMB267
Cry1Ac1	CP010092.1	113796	115895	723	CTTCATCAATAGGTGAAAATATCTATGCTTTT	100.0	Bacillus thuringiensis serovar galleriae	HD-29	pBMB126
**Bacillus thuringiensis serovar chinensis**									
Cry1Aa1	CP001910.1	264870	266969	723	CTTCATCAATAGGTGAAAATATCTATGCTTTT	100.0	Bacillus thuringiensis serovar chinensis	CT-43	pCT281
Cry1Ia1	CP001910.1	268907	271006	1781	TTTCACCTATCGATGAAAATATCTCTGCTTTT	100.0	Bacillus thuringiensis serovar chinensis	CT-43	pCT281
Cry2Ab1	CP001910.1	255124	257223	1300	CTTCTTCTATAGGTGAAGATATCTATGCTTTT	100.0	Bacillus thuringiensis serovar chinensis	CT-43	pCT281
**Bacillus thuringiensis serovar fukuokaensis**									
Cry9Da1	CP059144.1	100	2199	163	CTTCATCAATAGGTGAAAATATCTATGCTTTT	100.0	Bacillus thuringiensis serovar fukuokaensis	4AP1	pAP42
Cry9Da1	CP059143.1	76239	78338	163	CTTCATCAATAGGTGAAAATATCTATGCTTTT	100.0	Bacillus thuringiensis serovar fukuokaensis	4AP1	pAP258
Cry9Ee1	CP059143.1	52976	55075	79	CTTCATCAATAGGTGAAAATATGTATGCTTTT	100.0	Bacillus thuringiensis serovar fukuokaensis	4AP1	pAP258
**Bacillus thuringiensis server dendrolimus**									
Cry1Ab1	CP020005.1	328251	330350	723	TTTCATCAATGGGTGAAAATATCTATGCTTTT	100.0	Bacillus thuringiensis server dendrolimus	L-7601	unnamed3
Cry1Ab1	CP020005.1	347410	349509	723	CTTCATCAATAGGTGAAAATATCTATGCTTTT	100.0	Bacillus thuringiensis server dendrolimus	L-7601	unnamed3
Cry1Ia1	CP020005.1	332303	334402	1781	TTTCACCTATCGATGAAAATATCTCTGCTTTT	100.0	Bacillus thuringiensis server dendrolimus	L-7601	unnamed3
**Bacillus thuringiensis serovar morrisoni**									
Cry1Fb1	CP014852.1	82153	84252	766	CTTCATCAATAGGTGAAAATATCTATGCTTTT	100.0	Bacillus thuringiensis serovar morrisoni	HD12	pHD120161
Cry1Ja1	CP014853.1	145879	147978	707	CTTCATCAATAGGTGAAAATATCTATGCTTTT	100.0	Bacillus thuringiensis serovar morrisoni	HD12	pHD120345
Cry2Ad1	CP014853.1	201460	203559	797	CTTCTTCTATAGGTGAAGATATCTATGCTTTT	100.0	Bacillus thuringiensis serovar morrisoni	HD12	pHD120345
**Bacillus thuringiensis serovar alesti**									
Cry1Ae1	CP015177.1	228287	230386	888	CTTCATCAATAGGTGAAAATATCTATGCTTTT	100.0	Bacillus thuringiensis serovar alesti	BGSC 4C1	pBMB267
Cry2Ab1	CP015177.1	218770	220869	1302	CTTCTTCTATAGGTGAAGATATCTATGCTTTT	100.0	Bacillus thuringiensis serovar alesti	BGSC 4C1	pBMB267
**Bacillus thuringiensis serovar hailuosis**									
Cry1Ac1	CP013056.1	199500	201599	723	CTTCATGAATGGGTGAAAATATCTATGCTTTT	100.0	Bacillus thuringiensis serovar hailuosis	YWC2-8	pYWC2-8-1
Cry2Aa1	CP013056.1	232891	234990	25	CTTCCTCTATAGAGGAAGATATCTATGCTTTT	100.0	Bacillus thuringiensis serovar hailuosis	YWC2-8	pYWC2-8-1
**Bacillus thuringiensis serovar sumiyoshiensis**									
Cry9Da1	CP059150.1	251102	253201	163	CTTCATCAATAGGTGAAAATATCTATGCTTTT	100.0	Bacillus thuringiensis serovar sumiyoshiensis	4AO1	pAO254
Cry9Ee1	CP059150.1	227839	229938	79	CTTCATCAATAGGTGAAAATATGTATGCTTTT	100.0	Bacillus thuringiensis serovar sumiyoshiensis	4AO1	pAO254
**Bacillus thuringiensis serovar tolworthi**									
Cry1Ea1	AP014866.1	186315	188414	704	CTTCATGAATGGGTGAAAATATCTATGCTTTT	100.0	Bacillus thuringiensis serovar tolworthi	Pasteur Institute	pKK2
Cry1Ia1	AP014866.1	182296	184395	1781	TTTCACCTATCGATGAAAATATCTCTGCTTTT	100.0	Bacillus thuringiensis serovar tolworthi	Pasteur Institute	pKK2
**Bacillus thuringiensis serovar undetermined**									
Cry1Aa1	CP091111.1	52326	54425	723	CTTCATCAATAGGTGAAAATATCTATGCTTTT	100.0	Bacillus thuringiensis serovar undetermined	x023	unnamed1
Cry1Ac1	CP091111.1	3517	5616	723	CTTCATGAATGGGTGAAAATATCTATGCTTTT	100.0	Bacillus thuringiensis serovar undetermined	x023	unnamed1
Cry1Ia1	CP091111.1	48289	50388	1781	TTTCACCTATCGATGAAAATATCTCTGCTTTT	100.0	Bacillus thuringiensis serovar undetermined	x023	unnamed1
Cry2Aa1	CP091111.1	36908	39007	25	CTTCCTCTATAGAGGAAGATATCTATGCTTTT	100.0	Bacillus thuringiensis serovar undetermined	x023	unnamed1
Cry2Ab1	CP091111.1	62072	64171	1300	CTTCTTCTATAGGTGAAGATATCTATGCTTTT	100.0	Bacillus thuringiensis serovar undetermined	x023	unnamed1
Cry1Aa1	CP003755.1	9810	11909	723	CTTCATGAATGGGTGAAAATATCTATGCTTTT	100.0	Bacillus thuringiensis serovar undetermined	HD-771	p03
Cry1Aa1	CP170207.1	32231	34330	723	CTTCATCAATAGGTGAAAATATCTATGCTTTT	100.0	Bacillus thuringiensis serovar undetermined	B3	pBT-2
Cry1Ia1	CP170207.1	37934	40033	1781	TTTCACCTATCGATGAAAATATCTCTGCTTTT	100.0	Bacillus thuringiensis serovar undetermined	B3	pBT-2
Cry2Ab1	CP170207.1	22485	24584	1300	CTTCTTCTATAGGTGAAGATATCTATGCTTTT	100.0	Bacillus thuringiensis serovar undetermined	B3	pBT-2
Cry1Aa1	CP130745.1	232168	234267	723	CTTCATCAATAGGTGAAAATATCTATGCTTTT	100.0	Bacillus thuringiensis serovar undetermined	PS3	psecond
Cry1Ia1	CP130745.1	237872	239971	1781	TTTCACCTATCGATGAAAATATCTCTGCTTTT	100.0	Bacillus thuringiensis serovar undetermined	PS3	psecond
Cry2Ab1	CP130745.1	222422	224521	1300	CTTCTTCTATAGGTGAAGATATCTATGCTTTT	100.0	Bacillus thuringiensis serovar undetermined	PS3	psecond
Cry1Aa1	CP011350.1	611433	613532	723	CTTCATCAATAGGTGAAAATATCTATGCTTTT	100.0	Bacillus thuringiensis serovar undetermined	YC-10	pYC1
Cry1Ac1	CP011350.1	562625	564724	723	CTTCATGAATGGGTGAAAATATCTATGCTTTT	100.0	Bacillus thuringiensis serovar undetermined	YC-10	pYC1
Cry1Ia1	CP011350.1	607396	609495	1781	TTTCACCTATCGATGAAAATATCTCTGCTTTT	100.0	Bacillus thuringiensis serovar undetermined	YC-10	pYC1
Cry2Aa1	CP011350.1	596015	598114	25	CTTCCTCTATAGAGGAAGATATCTATGCTTTT	100.0	Bacillus thuringiensis serovar undetermined	YC-10	pYC1
Cry2Ab1	CP011350.1	621179	623278	1300	CTTCTTCTATAGGTGAAGATATCTATGCTTTT	100.0	Bacillus thuringiensis serovar undetermined	YC-10	pYC1
Cry1Ac1	CP155562.1	6984	9083	723	CTTCATGAATGGGTGAAAATATCTATGCTTTT	100.0	Bacillus thuringiensis serovar undetermined	HSY204	pC
Cry1Ac1	CP170208.1	15369	17468	723	CTTCATGAATGGGTGAAAATATCTATGCTTTT	100.0	Bacillus thuringiensis serovar undetermined	B3	pBT-3
Cry1Ac1	CP106948.1	16849	18948	723	CTTCATCAATAGGTGAAAATATCTATGCTTTT	100.0	Bacillus thuringiensis serovar undetermined	4F5	p_4F5-1
Cry1Fb1	CP076540.1	88903	91002	766	CTTCATCAATAGGTGAAAATATCTATGCTTTT	100.0	Bacillus thuringiensis serovar undetermined	GR007	pGR157
Cry1Ja1	CP076541.1	32294	34393	707	CTTCATCAATAGGTGAAAATATCTATGCTTTT	100.0	Bacillus thuringiensis serovar undetermined	GR007	pGR340
Cry2Ad1	CP076541.1	319233	321332	797	CTTCTTCTATAGGTGAAGATATCTATGCTTTT	100.0	Bacillus thuringiensis serovar undetermined	GR007	pGR340

Table S4: Genomic analysis of *ami* neighborhood

window_start	window_end	NCBI_Accession	NCBI_Organism	NCBI_plasmid	NCBI_strain	pident	evalue	bitscore	Cry name
**Bacillus thuringiensis serovar kurstaki**									
**229652**	240608	CP004876.1	Bacillus thuringiensis serovar kurstaki	pBMB299	HD1	99.745	0.0	2429	Cry1Aa
**86187**	95983	CP004875.1	Bacillus thuringiensis serovar kurstaki	pBMB95	HD1	85.376	0.0	2043	Cry1Ac
**47927**	58883	CP004873.1	Bacillus thuringiensis serovar kurstaki	pBMB65	HD1	90.025	0.0	2168	Cry1Ab
**8561**	19517	CP158704.1	Bacillus thuringiensis serovar kurstaki	unnamed6	HD-1	90.025	0.0	2168	Cry1Ab
**117969**	128925	CP158700.1	Bacillus thuringiensis serovar kurstaki	unnamed2	HD-1	99.830	0.0	2430	Cry1Aa
**69160**	80116	CP158700.1	Bacillus thuringiensis serovar kurstaki	unnamed2	HD-1	85.376	0.0	2043	Cry1Ac
**138154**	149110	CP116317.1	Bacillus thuringiensis serovar kurstaki	pLip300	Lip	99.745	0.0	2430	Cry1Aa
**172047**	183003	CP116317.1	Bacillus thuringiensis serovar kurstaki	pLip300	Lip	78.828	0.0	1664	Cry1Aa
**71107**	82063	CP116324.1	Bacillus thuringiensis serovar kurstaki	pLip97	Lip	90.025	0.0	2168	Cry1Ab
**11697**	22653	CP116324.1	Bacillus thuringiensis serovar kurstaki	pLip97	Lip	85.220	0.0	2045	Cry1Ac
**114886**	125842	CP009999.1	Bacillus thuringiensis serovar kurstaki	unnamed2	HD 1	99.830	0.0	2430	Cry1Aa
**66078**	77034	CP009999.1	Bacillus thuringiensis serovar kurstaki	unnamed2	HD 1	85.376	0.0	2043	Cry1Aa
**47152**	58108	CP010003.1	Bacillus thuringiensis serovar kurstaki	unnamed6	HD 1	90.025	0.0	2168	Cry1Aa
**201216**	212172	CP007615.1	Bacillus thuringiensis serovar kurstaki	pBMB293	YBT-1520	99.745	0.0	2430	Cry1Aa
**228081**	239037	CP004861.1	Bacillus thuringiensis serovar kurstaki	pBMB293	YBT-1520	99.745	0.0	2430	Cry1Aa
**39397**	50353	CP007613.1	Bacillus thuringiensis serovar kurstaki	pBMB69	YBT-1520	85.304	0.0	2046	Cry1Ac
**101333**	112289	CP072692.1	Bacillus thuringiensis serovar kurstaki	pKBT365	IMBL-B9	99.745	0.0	2430	Cry1Aa
**39937**	50893	CP072694.1	Bacillus thuringiensis serovar kurstaki	pKBT91	IMBL-B9	72.889	0.0	1709	Cry1Ea
**37329**	48285	CP083118.1	Bacillus thuringiensis serovar kurstaki	pCH_164-b	SA11	99.830	0.0	2430	Cry1Aa
**15592**	26548	CP083120.1	Bacillus thuringiensis serovar kurstaki	pCH_164-d	SA11	90.110	0.0	2169	Cry1Ab
**109788**	120744	CP083103.1	Bacillus thuringiensis serovar kurstaki	pCH_183-b	ABTS-351	99.830	0.0	2430	Cry1Aa
**14316**	25272	CP083107.1	Bacillus thuringiensis serovar kurstaki	pCH_183-f	ABTS-351	90.110	0.0	2169	Cry1Ab
**60979**	71935	CP083103.1	Bacillus thuringiensis serovar kurstaki	pCH_183-b	ABTS-351	85.376	0.0	2043	Cry1Ac
**2338**	13294	CP004070.1	Bacillus thuringiensis serovar kurstaki	pHT73	HD73	85.220	0.0	2045	Cry1Ac
**Bacillus thuringiensis serovar aizawai**									
**285831**	296787	CP116329.1	Bacillus thuringiensis serovar aizawai	pBLB1_317	BLB1	99.830	0.0	2430	Cry1Aa
**17316**	28272	CP116329.1	Bacillus thuringiensis serovar aizawai	pBLB1_317	BLB1	85.376	0.0	2043	Cry1Ac
**38564**	49520	CP059978.1	Bacillus thuringiensis serovar aizawai	pWK295	G03	99.695	0.0	2030	Cry1Aa
**97858**	108814	CP083146.1	Bacillus thuringiensis serovar aizawai	pCH_186-b	GC-91	99.695	0.0	2030	Cry1Aa
**20332**	31288	CP083147.1	Bacillus thuringiensis serovar aizawai	pCH_186-c	GC-91	85.220	0.0	2045	Cry1Ac
**245016**	255972	CP083087.1	Bacillus thuringiensis serovar aizawai	pP05_2-b	B401	99.695	0.0	2030	Cry1Aa
**15474**	26430	CP083091.1	Bacillus thuringiensis serovar aizawai	pP05_2-f	B401	90.025	0.0	2168	Cry1Ab
**13591**	24547	CP083161.1	Bacillus thuringiensis serovar aizawai	pCH_181-e	ABTS-1857	90.025	0.0	2168	Cry1Ab
**101420**	112376	CP083158.1	Bacillus thuringiensis serovar aizawai	pCH_181-b	ABTS-1857	99.745	0.0	2430	Cry1Aa
**1**	9928	DQ991096.1	Bacillus thuringiensis serovar aizawai	pCryAb1	SP-01-41	90.025	0.0	2168	Cry1Ab
**Bacillus thuringiensis serovar thuringiensis**									
**264953**	275908	CP004136.1	Bacillus thuringiensis serovar thuringiensis	pIS56-285	IS5056	99.745	0.0	2430	Cry1Aa
**4183**	15139	CP004131.1	Bacillus thuringiensis serovar thuringiensis	pIS56-63	IS5056	89.941	0.0	2164	Cry1Ab
**198646**	209602	CP043233.1	Bacillus thuringiensis serovar thuringiensis	unnamed2	Bt-GS57	99.490	0.0	2425	Cry1Aa
**4850**	15806	CP020758.1	Bacillus thuringiensis serovar thuringiensis	pLDW-19	ATCC 10792	79.858	0.0	663	Cry1Ab
**28031**	38987	CP021065.1	Bacillus thuringiensis serovar thuringiensis	poh4	ATCC 10792	90.025	0.0	2168	Cry1Ab
**Bacillus thuringiensis serovar galleriae**									
**242273**	253229	CP010091.1	Bacillus thuringiensis serovar galleriae	pBMB267	HD-29	99.745	0.0	2430	Cry1Aa
**109085**	120041	CP010092.1	Bacillus thuringiensis serovar galleriae	pBMB126	HD-29	85.220	0.0	2045	Cry1Ac
**Bacillus thuringiensis server dendrolimus**									
**342699**	353655	CP020005.1	Bacillus thuringiensis server dendrolimus	unnamed3	L-7601	87.764	0.0	1271	Cry1Ab
**324105**	335061	CP020005.1	Bacillus thuringiensis server dendrolimus	unnamed3	L-7601	90.363	0.0	2203	Cry1Ab
**Bacillus thuringiensis serovar chinensis**									
**260724**	271680	CP001910.1	Bacillus thuringiensis serovar chinensis	pCT281	CT-43	99.745	0.0	2430	Cry1Aa
**Bacillus thuringiensis serovar tolworthi**									
**181623**	192579	AP014866.1	Bacillus thuringiensis serovar tolworthi	pKK2	Pasteur Institute Stand	74.641	0.0	1759	Cry1Aa
**Bacillus thuringiensis serovar fukuokaensis**									
**48186**	59142	CP059143.1	Bacillus thuringiensis serovar fukuokaensis	pAP258	4AP1	55.076	0.0	1186	Cry9Ee
**Bacillus thuringiensis serovar sumiyoshiensis**									
**223049**	234005	CP059150.1	Bacillus thuringiensis serovar sumiyoshiensis	pAO254	4AO1	55.161	0.0	1188	Cry9Ee
**Bacillus thuringiensis serovar hailuosis**									
**194789**	205745	CP013056.1	Bacillus thuringiensis serovar hailuosis	pYWC2-8-1	YWC2-8	85.376	0.0	2043	Cry1Ac
**Bacillus thuringiensis serovar undetermined**									
**606722**	617678	CP011350.1	Bacillus thuringiensis serovar undetermined	pYC1	YC-10	99.830	0.0	2430	Cry1Aa
**557914**	568870	CP011350.1	Bacillus thuringiensis serovar undetermined	pYC1	YC-10	85.376	0.0	2043	Cry1Ac
**201211**	212167	ON470185.1	Bacillus thuringiensis serovar undetermined	pSY27.1_11	SY27.1	99.745	0.0	2430	Cry1Aa
**39911**	50867	ON470183.1	Bacillus thuringiensis serovar undetermined	pSY27.1_9	SY27.1	85.220	0.0	2045	Cry1Ac
**39789**	50745	ON470182.1	Bacillus thuringiensis serovar undetermined	pSY27.1_8	SY27.1	85.642	0.0	2055	Cry1Ac
**2338**	13294	ON470184.1	Bacillus thuringiensis serovar undetermined	pSY27.1_10	SY27.1	85.220	0.0	2045	Cry1Ac
**47615**	58571	CP091111.1	Bacillus thuringiensis serovar undetermined	unnamed1	x023	99.830	0.0	2430	Cry1Aa
**1**	9762	CP091111.1	Bacillus thuringiensis serovar undetermined	unnamed1	x023	85.314	0.0	2031	Cry1Ac
**11223**	22179	CP170208.1	Bacillus thuringiensis serovar undetermined	pBT-3	B3	85.220	0.0	2045	Cry1Ac
**28085**	39041	CP170207.1	Bacillus thuringiensis serovar undetermined	pBT-2	B3	99.695	0.0	2030	Cry1Aa
**2273**	13229	CP155562.1	Bacillus thuringiensis serovar undetermined	pC	HSY204	85.220	0.0	2045	Cry1Ac
**228022**	238978	CP130745.1	Bacillus thuringiensis serovar undetermined	psecond	PS3	99.695	0.0	2030	Cry1Aa
**4754**	15710	ON470192.1	Bacillus thuringiensis serovar undetermined	pSY62.1A_4	SY62.1A	89.941	0.0	2164	Cry1Ab
**86191**	95988	CP094406.1	Bacillus thuringiensis serovar undetermined	p95988	UFT038	85.642	0.0	2050	Cry1Ac
**256436**	267392	CP089522.1	Bacillus thuringiensis serovar undetermined	pB130-1	ZZQ-130	99.745	0.0	2430	Cry1Aa
**5099**	16055	CP003755.1	Bacillus thuringiensis serovar undetermined	p03	HD-771	97.966	0.0	2390	Cry1Aa
**12703**	23659	CP106948.1	Bacillus thuringiensis serovar undetermined	p_4F5-1	4F5	85.292	0.0	2041	Cry1Ac
